# Polymeric Nanocarriers of a Monosubstituted Tetraphenylporphyrin Sensitizer Intended for Photodynamic Therapy and Tumor Imaging

**DOI:** 10.1021/acsomega.5c07910

**Published:** 2025-11-10

**Authors:** Alžběta Turnovská, Jan Hynek, Marina Rodrigues Tavares, Muhammed Arshad Thottappali, Shanghui Gao, Volodymyr Lobaz, Jiří Pfleger, Jun Fang, Kamil Lang, Tomáš Etrych

**Affiliations:** † Institute of Macromolecular Chemistry, 86879 Czech Academy of Sciences, Prague 162 00, Czech Republic; ‡ Institute of Inorganic Chemistry, Czech Academy of Sciences, Řež 1001, Husinec-Řež 250 68, Czech Republic; § Laboratory of Microbiology and Oncology, Faculty of Pharmaceutical Sciences, 67181 Sojo University, Kumamoto 860-0082, Japan

## Abstract

To effectively combat advanced cancers, next-generation nanomedicines should combine both therapeutic and diagnostic functions. In this study, we developed stimulus-responsive theranostics systems based on micellar nanostructures that deliver derivatives of tetraphenylporphyrins (TPP) bound via tumor microenvironment-sensitive hydrazone bonds. These nanomedicines are engineered using a micelle-forming polymer-TPP conjugate, enabling the pH-sensitive activation of both photodynamic therapy (PDT) and fluorescence. Two pH-sensitive and one stable polymer-TPP conjugates were synthesized and characterized by size exclusion chromatography and TPP release rates. Micelle stability was evaluated using UV/vis spectroscopy, while fluorescence and singlet oxygen production were measured to determine their theranostics potential. Femtosecond transient absorption and time-correlated single photon counting techniques were employed for the photophysical evaluation of micellar systems. Compared to polymer conjugates where TPP is linked through nondegradable amide bonds, the pH-sensitive systems exhibit superior physicochemical properties. These micellar conjugates are highly stable, allowing prolonged circulation in the body while remaining in an “off” state, where fluorescence and singlet oxygen production are minimized. Overall, the hydrazone-linked conjugates display favorable properties that make them strong candidates for future anticancer theranostic applications.

## Graphical abstract

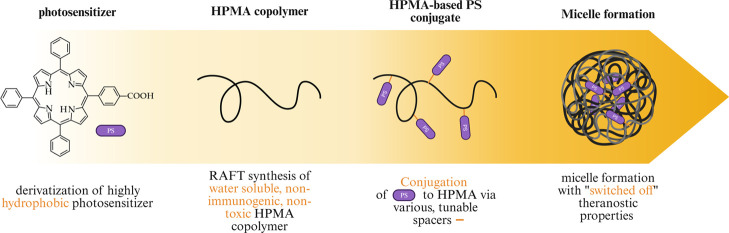

## 1 Introduction

Photodynamic therapy (PDT) is a treatment modality employed to treat various malignant tumors. It is a clinically used therapeutic procedure that has gained attention since several drugs were approved by regulatory authorities for the treatment of bladder and esophageal cancer in 1993 and 1994, respectively.^1^ Since then, PDT has found its way into clinical trials of other malignant tumors, including lung cancer,^2–4^ head and neck cancer,^5–7^ prostate cancer,^8–11^ and skin cancer.^12–14^

PDT relies on light activation (with an appropriate wavelength) of a chemical compound called photosensitizer (PS) in the presence of molecular oxygen. Porphyrins are widely used as PSs for PDT because of their specific photochemical behavior, arising from the 18 π-electron aromatic macrocycle. More specifically, *meso*-substituted porphyrins, such as lipophilic 5,10,15,20-tetraphenylporphyrins (TPPs), are widely used in PDT, due to their relatively easy synthesis.^15,16^ They exhibit a characteristic absorption spectrum with a strong π–π* transition, the so-called Soret band, and four Q bands in the visible region. These bands and their position are essentially a reflection of the electronic transitions within the chromophore and are therefore structure-dependent and easily tunable.^17–19^

By absorbing the light, the PS in its ground state is excited into a short-lived singlet state, from which it can undergo intersystem crossing through spin conversion of an electron in the higher-energy orbital into a more stable and long-lived triplet state. Long lifetimes of the triplet state allow energy to be transferred onto surrounding molecules, including molecular oxygen, creating reactive oxygen species (ROS).^20^ Two mechanisms of PDT, based on where excited PS (PS*) transfers its energy, are known. A type I mechanism occurs when PS* interacts with biomolecules from its surroundings and involves hydrogen atom abstraction or electron transfer reactions, generating free radicals and radical ions, yielding ROS such as superoxide radical anion (O_2_^•–^), hydroxyl radical (OH^•^), and peroxide (H_2_O_2_). A type II mechanism involves direct energy transfer from PS* to molecular oxygen, creating singlet oxygen O_2_(^1^Δ_g_). Acute oxidative stress, induced by ROS, initiates a cascade of cytotoxic pathways resulting in apoptosis or necrosis. In addition, PDT provides the antitumor effect in three ways: (i) by direct cytotoxic effect on tumor cells, (ii) by destruction of surrounding vasculature, and (iii) by stimulation of antitumor immunity, offering a significant long-term benefit compared to immunosuppressive conventional therapies.^21^

Compared to the conventional cancer treatment, such as surgery, chemotherapy, and radiation, PDT offers lower invasiveness. Moreover, by illuminating only the affected area, we can precisely and directly target the tumor site, with minimal intervention to the surrounding healthy tissue. If necessary, it can be repeated multiple times at one location, unlike radiotherapy and surgery, leaving zero to minimal scarring.^22^ Despite these advantages, the use of conventional PSs for PDT is limited by their inherent hydrophobicity, low solubility in aqueous media, and susceptibility to aggregation. This naturally occur-ring self-assembly via noncovalent interactions can significantly alter the physicochemical properties of a PS due to self-quenching, resulting in reduced singlet oxygen production necessary for efficient PDT.^23–25^ Electronic transitions in TPP are mainly determined by the π-conjugated porphyrin ring. If the molecule is excited to higher singlet states, it relaxes to the *Q*_*x*_ within 100 fs after the photoexcitation. During about 100–200 fs, the lowest *Q*_*x*_ state undergoes an intramolecular vibrational energy redistribution, followed by the vibrational redistribution caused by elastic collisions with solvent within the next 1.4 ps and thermal equilibration of molecules via energy exchange with the solvent taking place in the time scale of 10–20 ps.^26^ The decay of the equilibrated *Q*_*x*_ state population occurs on the nanosecond time scale by fluorescence, but mainly, with the efficiency about 80%, via intersystem crossing to triplet T_1_ states with lifetime reaching milliseconds in degassed solvents.^27,28^ From the long-lived triplet states, the energy can be transferred to oxygen, forming the desired singlet oxygen. However, there are several competitive processes reported that limit the triplet state lifetime, such as phosphorescence, nonradiative relaxations, and also triplet–triplet annihilation that involves two molecules, the first is one excited back to the singlet state, and the second transferred to its ground state.^29^ These processes are strongly dependent on the local concentration of PS and its diffusion rate in solution and, in the case of aggregate formation, also on mutual interactions of PS molecules within the aggregate.

The above limitations of PDT can be overcome by the use of nanotechnology. There are various approaches currently under investigation to deliver hydrophobic PSs with low solubility. Liposomes, lipid nanoparticles, dendrimers, oil-dispersions, polymeric nano- or microparticles, or hydrophilic polymer-PS conjugates can be employed to increase the uptake and treatment efficiency in tumorous target tissue. Those systems are able to solubilize the PS and avoid the off-target toxicity related with free PS.^30^ The carrier should be able to incorporate or bind PS without altering its activity and ensuring its safety and high accumulation. The system should be biocompatible and have limited to no toxicity and immunogenicity.^31^ Moreover, these systems can preferentially accumulate inside the tumor tissue due to the enhanced permeability and retention (EPR) effect. The tumor’s leaky vasculature with a disrupted endothelial barrier and hence resulting increased permeability, as well as the underdeveloped and obstructed lymphatic system, are sufficient for the carriers to accumulate in the tumor by simple diffusion.^32,33^ Despite all of the improvements, PDT is still suitable only for treating localized cancers. Absorption of light by the biological tissues (f.e., hemoglobin) or scattering due to tissue heterogeneity constitutes another major limitation when it comes to treatment of tumors in the vicinity of blood vessels or deeply seated tumors.^34,35^

Among possible PS carriers, water-soluble and biocompatible systems based on *N*-(2-hydroxypropyl)­methacrylamide (HPMA) copolymers have been extensively utilized as promising candidates for cancer treatment. In fact, HPMA-drug conjugates have already been approved for clinical trials or have been used in compassionate use in the treatment of cancerous diseases.^36,37^ Recently, HPMA micellar conjugates bearing pyropheophorbide-a (PyFa) showed a remarkable antitumor effect with selective accumulation in the tumor and high O_2_(^1^Δ_g_) generation, making them promising candidates for theranostics.^38,39^ Despite advances in cancer therapy, there is still growing need for dual-function theranostic agents to overcome the disconnect between diagnosis and therapy, which can cause delays in treatment, increased systemic toxicity, and reduced overall efficacy. Dual-function theranostic agents are designed to integrate imaging and therapy in a single platform, addressing these limitations. Importantly, previously used PSs such as zinc protoporphyrin IX or PyFa do not reach the photocytotoxicity of the clinically used PDT agent temoporfin (mTHPC). Conversely, monosubstituted tetraphenyl porphyrins show comparable activity to that of mTHPC. Thus, these findings highlight the high therapeutic potential of TPP-COOH as a PDT agent in drug delivery systems.^39–41^

Herein, we present the syntheses, physicochemical characterizations, and PDT properties of HPMA-based polymer nanosystems bearing a TPP-COOH photosensitizer. We aimed to solubilize water-insoluble TPP-COOH molecules by incorporating them into biocompatible micelle-forming HPMA copolymers, thus protecting and prolonging their blood circulation during transport, while maintaining the inherent physical behavior of the TPP-COOH photosensitizer which is necessary to elicit cell death. TPP-COOH molecules were connected to the copolymer backbone either by stable amide bonds or by hydrolytically labile hydrazone bonds, with two types of spacers, i.e., aliphatic (5-hydroxy-2-pentanone) and aromatic (1-(4-hydroxymethyl)­phenyl)­ethanone). Polymer conjugates were afterward characterized to determine their applicability as future polymer nanomedicines serving for both the therapy and visualization of the tumors.

## 2 Materials and Methods

### 2.1 Chemicals

2,2′-Azobis­(isobutyronitrile) (AIBN), methacroyl chloride, 1-aminopropan-2-ol, 6-aminohexanoic acid, *N*-(3-(dimethylamino)­propyl)-*N*′-ethylcarbodiimide hydrochloride (EDC), carbon disulfide, ethanethiol, sodium hydride (60% dispersion in mineral oil), 5-hydroxy-2-pentanone, 2-thiazoline-2-thiol (TT), 4,6-trinitrobenzene-1-sulfonic acid (TNBSA), 4-(dimethylamino)­pyridine (DMAP), *N*,*N*-diisopropylethylamine (DIPEA), 4,4′-azobis­(4-cyanovaleric acid) (ACVA), 4-(2-carboxyethylsulfanylcarbothioylsulfanyl)-4-cyanopentanoic acid (carboxyethyl-TTc-ACVA), *tert*-butanol (*t*-BuOH), *N*,*N*-dimethylacetamide (DMA), dimethyl sulfoxide (DMSO), dichloromethane (DCM), 1,4-dioxane, benzaldehyde, Tween-20, lithium bromide (LiBr), and sodium dodecyl sulfate (SDS) were obtained from Merck (Czech Republic). 1-(4-Hydroxymethyl)­phenyl)­ethanone was obtained from abcr GmbH (Germany). *N*-(3-*tert*-Butoxycarbonyl-aminopropyl)­methacrylamide (Ma-AP-NH-Boc) was obtained from Polysciences, Inc. (USA). Methanol (MeOH), acetic acid, propionic acid, chloroform (CHCl_3_), hydrochloric acid (HCl), *N*-hexane, and ethyl acetate were obtained from Lach:Ner (Czech Republic), pyrrole from FluoroChem (UK), and methyl 4-formylbenzoate from BLDpharm (Germany). Azoinitiator 2,2′-azobis­(4-methoxy-2,4-dimethylvaleronitrile) (V-70) was obtained from Wako Pure Chemical Industries Ltd. (Japan). Trifluoroacetic acid (TFA) was purchased from Iris Biotech GmbH (Germany). All the solvents used were of analytical grade. Ethanol (EtOH), *N*,*N*-dimethylformamide (DMF), and acetonitrile, as well as the solvents for the nuclear magnetic resonance (NMR) characterization of DMSO-*d*_6_ (99.80 atom % D) and CDCl_3_ (99.80 atom % D) were obtained from VWR Chemicals (Belgium). Column chromatography was performed on silica gel (0.060–0.200 mm, 60 Å) purchased from Thermo Scientific (Czech Republic). Acetate-TT was synthesized by means of carbodiimide chemistry from acetic acid (2.0 g, 33.3 mmol) by reaction with 2-thiazoline-2-thiol (4.2 g, 35.0 mmol) in the presence of EDC (8.3 g, 43.3 mmol) in 20 mL of DCM for 2 h. Extraction with water and NaHCO_3_ was used as the purification step; the organic phase containing acetate-TT was dried with Na_2_SO_4_ and then allowed to crystallize in the freezer. ^1^H NMR (400 MHz, DMSO-*d*_6_): 4.48 (t, *J* = 7.6 Hz, 2H, –N–CH_2_–CH_2_–S/-N–CH_2_–CH_2_–S–); 3.37 (t, *J* = 7.6 Hz, 2H, –N–CH_2_–CH_2_–S/-N–CH_2_–CH_2_–S-); 2.65 (s, 3H, CH_3_–CO−).

### 2.2 Synthesis of 5,10,15-Triphenyl-20-(4-carboxyphenyl) porphyrin (TPP-COOH)

The title compound was synthesized using a previously published method of statistic condensation.^42^ A mixture of 11.0 mL (108 mmol) of benzaldehyde and 5.92 g (36 mmol) of methyl 4-formylbenzoate was dissolved in 500 mL of propionic acid and heated to 140 °C. After that, 10 mL (144 mmol) of pyrrole was added, and the solution was stirred at the set temperature for 4 h. After being cooled, the formed purple precipitate was collected by filtration and washed with methanol. From the obtained mixture of products, the target ester of the monocarboxylic porphyrin was separated using column chromatography on silica gel, using CH_2_Cl_2_ as eluent. The methyl ester of the target product was prepared in 1.51 g (6%) yield. The purity of the product was determined by ^1^H NMR spectroscopy and high-resolution mass spectrometry (HRMS). ^1^H NMR (600 MHz, 295 K, CDCl_3_): δ = 8.88–8.83 (m, 6H, Ar), 8.79 (d, 2H, *J* = 4.7 Hz, Ar), 8.44 (d, 2H, *J* = 8.2 Hz, Ar), 8.30 (d, 2H, *J* = 8.2 Hz, Ar), 8.21 (dt, *J* = 6.3, 1.6 Hz, 6H, Ar), 7.81–7.69 (m, 9H, Ar), 4.11 (s, 3H, –OCH_3_), −2.79 (s, 2H, –NH−) ppm. HRMS calcd for C_46_H_33_N_4_O_2_ 673.2598, found 673.2560. In the following reaction step, 600 mg of the prepared ester was dissolved in 120 mL of THF, 30 mL of 1 M NaOH water solution was added, and the mixture was refluxed for 16 h. After cooling down, THF was removed by rotary evaporation, and the precipitate formed in the residue was collected by centrifugation (Hettich Rottina 380R, 11,000 rpm, 10 min) and thoroughly washed with 0.1 M HCl and water. The resulting purple product was dried in vacuo. The target compound was prepared in 540 mg (92%) yield. ^1^H NMR (600 MHz, 295 K, DMSO-*d*_6_): δ = 8.80 (s, 8H, Ar), 8.34 (d, 2H, *J* = 8.1 Hz, Ar), 8.29 (d, 2H, *J* = 8.1 Hz, Ar), 8.19 (dt, *J* = 6.0, 1.7 Hz, 6H, Ar), 7.85–7.76 (m, 9H, Ar), −2.96 (s, 2H, –NH−) ppm. HRMS calcd for C_45_H_31_N_4_O_2_ 659.2442, found 659.2406.

### 2.3 Synthesis of Monomers and CTA

*N*-(2-hydroxypropyl)­methacrylamide (HPMA) was synthesized via the reaction of methacryloyl chloride with 1-aminopropan-2-ol in DCM with the presence of sodium hydrogen carbonate as described in the literature.^43^ *N*-(*tert*-butoxycarbonyl)-*N*′-(6-methacrylamidohexanoyl)­hydrazine (MA-Ahx-NHNH-Boc) was prepared using a two-step synthesis following the method for synthesis of Ma-Ahx-COOH described elsewhere.^44^ The trithiocarbonate chain transfer agent *S*-2-cyano-2-propyl-*S*′-ethyl trithiocarbonate (AIBN-TTc) was synthesized as described by Ishitake et al.^45^

### 2.4 Synthesis of Polymer Precursors

The polymer precursor **P1** p­(HPMA-*co*-Ma-Ahx-NHNH_2_) with hydrazide groups alongside the main chain was prepared via the controlled radical reversible addition–fragmentation chain transfer (RAFT) polymerization of HPMA and Ma-Ahx-NHNH-Boc monomers, with a 95/5 molar ratio, in a 80/20 (*v*/*v*) *t*-BuOH/DMA mixture, in the presence of V-70 initiator and AIBN-TTc chain transfer agent (CTA). The monomer/CTA/initiator molar ratio was set at 225/1/0.5. The reaction was carried out as follows: HPMA (2.0 g, 14.0 mmol) and Ma-Ahx-NHNH-Boc (0.2 g, 0.7 mmol) were dissolved in *t*-BuOH (16.8 mL), while AIBN-TTc (13.4 mg, 65.3 μmol) and V-70 (10.1 mg, 32.7 μmol) were dissolved in DMA (4.2 mL). Both solutions were mixed inside an ampule, bubbled for 10 min with argon, and sealed. After 72 h at 30 °C, the mixture was precipitated into acetone/diethyl ether (2/1), and the precipitated polymer was filtered off and dried under vacuum. The yield was 1.9 g (83%). For the removal of TTc groups, **P1** (1.9 g) and AIBN (370.2 mg, 2.3 mmol) were dissolved in DMA (14.8 mL), inserted into an ampule, bubbled with argon (10 min), and left at 80 °C for 3 h. The polymer was obtained by precipitation into acetone/diethyl ether (2/1), filtered out, and dried under vacuum. Hydrazide groups’ Boc-deprotection was carried out by dissolving **P1** (1.7 g) in Q-water (1.7 mL), bubbling with argon (10 min) inside an ampule, and boiling at 100 °C for 1 h. The final polymer precursor was obtained by lyophilization. No further purification was needed. The content of the hydrazide groups was evaluated using UV/vis spectrophotometry.

Polymer precursor **P2** p­(HPMA-*co*-Ma-AP-NH_2_) with amine groups alongside the main chain was prepared analogously using RAFT polymerization of HPMA and Ma-Ap-NH-Boc with the ACVA initiator and carboxyethyl-TTc-ACVA CTA. The reaction was carried out in a distilled water/1,4-dioxane mixture (2/1) at 70 °C for 7 h. The monomers/CTA/initiator ratio was 225/1/0.5, and the ratio of monomers HPMA/Ma-Ap-NH-Boc was 96/4. The TTc end-group removal was carried out via a reaction with an excess of AIBN at 80 °C for 3 h in DMA, and amine groups were thermally deprotected in Q-water at 150 °C for 1.5 h. The final polymer precursor was obtained by lyophilization. Content of the amine groups was determined using UV/vis spectrophotometry. The characterization of both **P1** and **P2** polymer precursors is summarized in Table 1. The detailed synthesis scheme is depicted in Figure 1a.

**Table 1:** Physicochemical Characterization of Polymer Precursors^a^

precursor	FG^b^	content of FG [mol %]	*M*_n_ [g mol^–1^]	*M*_w_ [g mol^–1^]	*Đ*	*D*_H_ ± SD [nm]
**P1**	hydrazide	4.5	34,600	36,400	1.05	9.5 ± 0.3
**P2**	amine	4.0	36,120	39,300	1.09	7.4 ± 0.1

a *M*_n_ is the number-average molecular weight, *M*_w_ is the weight-average molecular weight, *Đ* is the dispersity, and *D*_H_ is the hydrodynamic diameter measured by DLS.

b FG means functional group.

**Figure 1:**
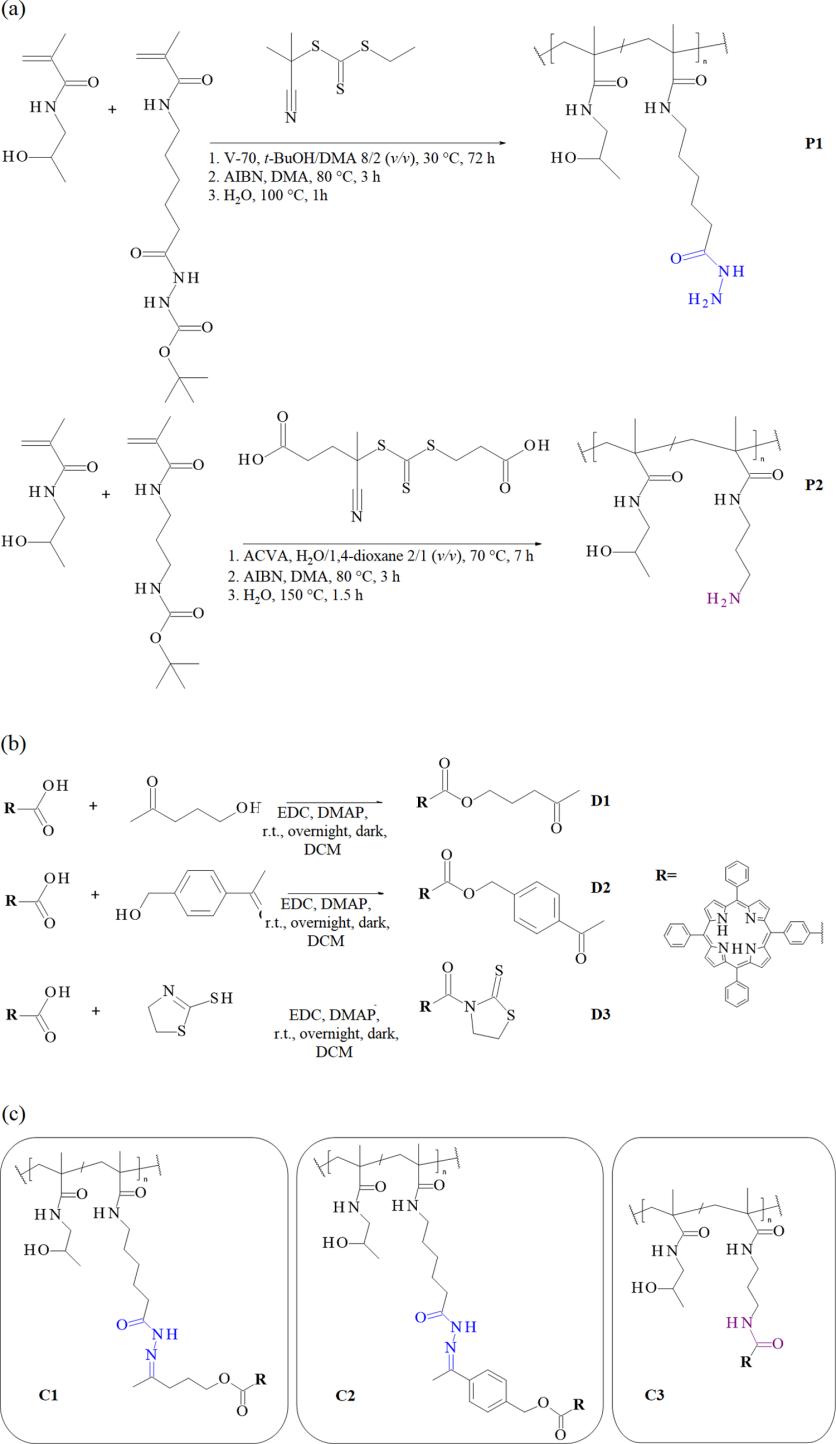
Scheme of the synthesis of (a) polymer precursors **P1** and **P2** and (b) TPP-COOH derivatives **D1-D3**, and (c) structures of final polymer/photosensitizer conjugates **C1**–**C3**.

### 2.5 Synthesis of Porphyrin Derivatives

Carbodiimide chemistry was used for the transformation of the TPP-COOH to its derivatives (dTPP) suitable for the formation of hydrazone/amide bonds with the polymer backbone. Derivate **D1** with an aliphatic spacer between the TPP and keto group was prepared by the reaction of TPP-COOH (50.0 mg, 75.9 μmol) with EDC (21.8 mg, 113.8 μmol), 5-hydroxy-2-pentanone (11.6 mg, 113.8 μmol), and DMAP. Similarly, derivate **D2** with an aromatic spacer was obtained via mixing TPP-COOH (50.0 mg, 75.9 μmol) with EDC (21.8 mg, 113.8 μmol), 1-(4-hydroxymethyl)­phenyl)­ethanone (17.1 mg, 113.8 μmol), and DMAP. Derivate **D3** with the carboxyl group converted to more reactive 2-thiazoline-2-thiol amide was prepared from TPP-COOH (50.0 mg, 75.9 μmol) and EDC (21.8 mg, 113.8 μmol) dissolved in DCM. After 30 min of stirring, 2-thiazoline-2-thiol (TT) (4.6 mg, 38.9 μmol) and DMAP catalyst were added. The preparation of all the derivatives (**D1**–**D3**) was carried out in DCM using continuous stirring overnight at room temperature in the dark. The reactions were monitored using TLC with hexane/ethyl acetate 2/1 (*v*/*v*) as a mobile phase, and the products were purified by silica gel (60 Å) column chromatography with a gradient of the hexane/ethyl acetate mobile phase with a gradient from 7/1 to 1/1 (*v*/*v*). The derivatives **D1**–**D3** were characterized by ^1^H NMR and MALDI analysis. The detailed MALDI methodology and spectra (Figure S1) can be found in the Supporting Information. D1: ^1^H NMR (600 MHz, 295 K, DMSO-*d*_6_): δ = 9.02–7.65 (m, 27H, Ar), 4.43 (t, 2H, *J* = 6.3 Hz, –OCH_2_–CH_2_−), 2.75 (t, 2H, *J* = 7.0 Hz, –CH_2_–CH_2_–C­(O)−), 2.18 (s, 3H, –CH_3_), 2.04 (p, 2H, *J* = 6.7 Hz, –CH_2_–CH_2_–CH_2_−), −2.93 (s, 2H, –NH−) ppm. **D2**: ^1^H NMR (600 MHz, 295 K, DMSO-*d*_6_): δ = 9.03–7.63 (m, 31H, Ar), 5.62 (s, 2H, –CH_2_−), 2.62 (s, 3H, –CH_3_), −2.93 (s, 2H, –NH−) ppm. **D3**: ^1^H NMR (400 MHz, 300 K, DMSO-*d*_6_): δ = 9.12–7.61 (m, 27H, Ar), 4.70 (t, 2H, *J* = 7.1 Hz, –CH_2_–CH_2_−), 3.73 (t, 2H, *J* = 7.1 Hz, –CH_2_–CH_2_−), −2.92 (s, 2H, –NH−) ppm. The detailed reaction schemes are shown in Figure 1b.

### 2.6 Synthesis of Conjugates

Polymer precursor **P1** with hydrazide groups was used for the attachment of **D1** and **D2** derivatives, forming conjugates with degradable hydrazone bonds, **C1** and **C2**, with an aliphatic and aromatic spacer, respectively. As an example, polymer **P1** (350.0 mg) and derivative **D2** (35.0 mg, 44.2 μmol) were dissolved in 3.5 mL of MeOH/DMA 8/2 (*v*/*v*), 525.0 μL of acetic acid was added, and the reaction mixture was left stirring continuously at room temperature overnight in the dark. Conjugate **C2** was purified by three times repeated precipitation of the reaction mixture into an ethyl acetate/CHCl_3_ 5/2 (*v*/*v*) mixture. The precipitate was filtered off, washed with ethyl acetate/diethyl ether 1/1 (*v*/*v*), and dried under vacuum to obtain final conjugate **C2** (304 mg, yield 79%). Conjugate **C1** was obtained using the same procedure (308 mg, yield 88%). The purity of conjugates was assessed by TLC using a hexane/ethyl acetate 2/1 (*v*/*v*) mixture as a mobile phase.

Polymer precursor **P2** (140.0 mg) with amine groups and derivative **D3** (14.0 mg, 18.4 μmol) were mixed in 1.4 mL of a MeOH/DMA 8/2 (*v*/*v*) mixture with an excess of DIPEA (6.4 μL, 36.8 μmol) and stirred continuously overnight at room temperature in the dark. The purification procedure was analogous as mentioned above. To remove any residual, and potentially cytotoxic, amino-groups after **D3** attachment, the conjugate was further reacted with an excess of acetate-TT for 10 min, precipitated into ethyl acetate three times, and dried under vacuum, to obtain final conjugate **C3**.

Purity of all conjugates **C1**, **C2**, and **C3** was evaluated by TLC using a hexane/ethyl acetate 2/1 (*v*/*v*) mobile phase and by size exclusion chromatography. The structures of the final conjugates and the reaction schemes are presented in Figure 1c.

### 2.7 Dynamic Light Scattering (DLS)

Dynamic light scattering (Zetasizer Ultra, Malvern Panalytical, Great Britain) was used to measure the hydrodynamic diameters (*D*_H_) of polymer precursors **P1** and **P2** and polymer conjugates **C1**–**C3** at λ = 632.8 nm and θ = 173°. Fluorescence filtering was applied for the conjugates, and the Zetasizer “ZS XPLORER” software was used for the data evaluation. A fluorescence filter was applied for the conjugates. **P1** and **P2** were measured at 3.0 mg mL^–1^ in Q-water, while the conjugates **C1**–**C3** were investigated at 1.0 mg mL^–1^ in phosphate buffer (PB) pH 7.4/EtOH 5% (*v*/*v*). The data are displayed in Table 2.

**Table 2:** Physicochemical Characterization of Polymer Conjugates^a^

polymer conjugate	spacer	content of dTPP [wt %]	*D*_H_ ± SD [nm]
**C1**	5-hydroxy-2-pentanone	5.8 (**D1**)	16.4 ± 1.8
**C2**	1-(4-hydroxymethyl)phenyl)ethanone	5.8 (**D2**)	19.1 ± 0.5
**C3**	2-thiazoline-2-thiol	6.2 (TPP-COOH)	19.1 ± 2.0

a *D*_H_ is the hydrodynamic diameter measured by DLS in phosphate buffer pH 7.4/EtOH 5% (*v*/*v*) with concentration 1.0 mg mL^–1^ at 25 °C. A fluorescence filter was applied.

### 2.8 Size Exclusion Chromatography (SEC)

Size exclusion chromatography was used for the determination of number-average molecular weight (*M*_n_), weight-average molecular weight (*M*_w_), and dispersity (*Đ*) of polymer precursors **P1** and **P2** and for the determination of the purity of final conjugates **C1**–**C3**. The detailed SEC methodology can be found in the Supporting Information in Section 2.8.

### 2.9 UV–Vis Spectrophotometry

UV/vis spectrophotometry was used to calculate the molar content of hydrazide and amine groups alongside the polymer chain of **P1** and **P2** following the 2,4,6-trinitrobenzene-1-sulfonic acid (TNBSA) assay method, as described in the literature.^46^

The amount of bound porphyrin (wt %) was determined in the MeOH/DMA (8/2, *v*/*v*) mixture for **C1** and **C2**, and DMSO for **C3**, by using the corresponding molar absorption coefficients—393,900 M^–1^ cm^–1^ for **D1**, 426,300 M^–1^ cm^–1^ for **D2**, and 311,700 M^–1^ cm^–1^ for TPP-COOH, measured at λ = 416 nm.

### 2.10 Fluorescence Spectroscopy

The imaging potentials of micellar conjugates **C1**–**C3** were evaluated using fluorescence spectroscopy. The fluorescence emission spectra of TPP-COOH and all conjugates were recorded by a spectrofluorometer JASCO FP-6200, Tokyo, Japan, equipped with the Spectra Manager software. Conjugates **C1**–**C3** were dissolved in phosphate buffer pH 7.4 containing 5% (*v*/*v*) DMSO, with or without the presence of the indicated concentrations of Tween-20 or sodium dodecyl sulfate (SDS), as presented in the Supporting Information (Figure S4). For comparison, TPP-COOH was dissolved in DMSO. The solutions were prepared in concentrations equivalent to 5.0 μg mL^–1^ of TPP-COOH, excited at 514 nm, and the emission spectra were recorded within 550–800 nm. To determine the effect of the dTPP release on the fluorescence properties, the conjugates were incubated for 24 h in phosphate buffer pH 5.0 containing 5% DMSO (*v*/*v*) in the presence of 0.1% SDS.

### 2.11 Critical Micellar Concentration (CMC) Evaluation

The UV/vis spectra of conjugates **C1**–**C3** were measured in 10 mM PBS in the absence and presence of 10% human blood plasma in a wavelength range of 300–700 nm. The conjugate concentration ranged from 0.1 mg mL^–1^ to 48.0 ng mL^–1^, as concentrations higher and lower were not measurable. The measured spectra contained two overlapping Soret bands at 403 and 423 nm, corresponding to stacked and monomeric porphyrin molecules, respectively. The complex Soret band was deconvoluted by two Gaussian peaks. The ratio of peak area, proportional to the ratio of concentration of porphyrin aggregates (403 nm) and single molecules (423 nm), was plotted vs the logarithm of concentration (mg mL^–1^). For details about CMC evaluation, see the Supporting Information (Figure S2).

### 2.12 Isothermal Titration Calorimetry (ITC)

ITC was performed using a MicroCal ITC 200 instrument (Malvern Panalytical Ltd., UK). A solution of the respective polymer or polymer-porphyrin conjugate in PBS (4.0 mg mL^–1^) was titrated into human blood plasma, diluted 10-fold with PBS. For data analysis, the concentration of plasma proteins was expressed as 5.0 mg mL^–1^ human serum albumin (0.076 mM). Titrations were conducted at 25 °C. An initial injection of 0.2 μL was followed by 19 injections of 2.0 μL each. Data from the first injection were excluded from the analysis. Blank titrations in PBS were also performed, and the resulting isotherms were corrected for the corresponding heat of dilution using Affinimeter software version 1.2.3 (S4SD-AFFINImeter, Santiago de Compostela, Spain). The enthalpies were plotted as a function of the molar ratio of the polymer to serum albumin. The associated errors were estimated from the signal-to-noise ratio during software-based integration of the raw ITC heat flux versus time data.

### 2.13 Release of dTPP from the Polymer Conjugates **C1** and **C2**

The release of **D1** and **D2** was carried out by incubating conjugates **C1** and **C2** (at 2.0 mg mL^–1^), respectively, in 0.1 M phosphate buffer solutions at pH 5.0 and pH 7.4 at 37 °C, and DMSO 5% (*v*/*v*) was used for better dissolution of samples. At each time point, an aliquot of the sample was rigorously shaken with chloroform to extract the released derivative. The released amount was then dissolved in DMF and was determined by HPLC analysis. Measurements were performed in triplicates. For details of HPLC analysis of dTPP release, see Supporting Information Section 2.13.

Calibration curves using the relative area of peaks (absorbance at λ = 416 nm) corresponding to **D1** or **D2** at different concentrations were measured in triplicate and used later to determine dTPP release. The release was expressed relative to the total derivative content in the conjugate. The theoretical maximal amount of released **D1** or **D2**, calculated using the content of **D1** or **D2** in each conjugate, and measured calibration curve were in agreement with the released derivative after 4 h of incubation at pH 1.7.

### 2.14 Electron Spin Resonance Spectroscopy

The generation of the O_2_(^1^Δ_g_) was measured using electron spin resonance (ESR) spectroscopy. Conjugate **C1**–**C3** solutions were prepared with different solvents (PBS with different pHs, DMSO, and Tween-20) at 40 μg mL^–1^ (TPP-COOH eq). To 900 μL of the sample, 100 μL of 300 mM 2,2,6,6-tetramethyl-4-piperidone (TOKYO CHEMICAL INDUSTRY JAPAN) was added. The samples were then subjected to light irradiation using a xenon light source (MAX-303; Asahi Spectra Co., Ltd., Tokyo, Japan) with a wavelength of 400–700 nm, with 300 s of light irradiation, and the X-band ESR spectra were recorded using a JEOL JES FA-100 spectrometer (Tokyo, Japan) at 25 °C. The ESR spectrometer was set at a microwave power of 1.0 mW, amplitude of 100 kHz, and field modulation width of 0.1 mT.

### 2.15 In Vitro Cytotoxicity

Cytotoxicity of free TPP-COOH and conjugates **C1**–**C3** was evaluated on mouse colon cancer C26 cells using the MTT assay.^47^ The measurements were conducted in 96-well plates with 5000 cells per well. After 24 h of cultivation in RPMI-1640 with 10% fetal bovine serum (PBS, Nichirei Biosciences INC., Tokyo, Japan) at 37 °C under 5% CO_2_, the TPP-COOH and conjugates **C1**–**C3** were added at different concentrations. After 24 h of treatment, the medium was removed, and cells were washed by PBS several times and poured over with fresh medium. Illumination was carried out with blue light (λ = 420 nm, TL-D; Philips, Eindhoven, the Netherlands) at 1.0 J cm^–2^ for 5 min. After another 24 h of treatment, the viability of cells was quantified, and IC_50_ values were determined.

### 2.16 Fluorescence Lifetime

Fluorescence lifetime was measured with a time-correlated single photon counting (TCSPC) technique using a FluoTime 300 system (PicoQuant, Germany), equipped with a PicoHarp 300-Stand-alone TCSPC Module and LDH-P-C-390 laser diode emitting at a central wavelength of 389 nm. Alternatively, a tunable Solea White Supercontinuum laser excitation source was used, operating in the wavelength range 480–700 nm. The instrument response function (IRF) had a full-width at half-maximum of approximately 200 ps. The kinetics were measured on TPP-COOH and polymer conjugates’ solutions in PBS (pH 7.4) and in DMSO in the concentration range 25 to 100 μg mL^–1^, corresponding to the concentration of the TPP-COOH chromophores ∼1.5 × 10^–6^ to 6 × 10^–6^ mol L^–1^. Addition of a small amount of EtOH was necessary to dissolve TPP-COOH in PBS. The fluorescence decay was analyzed by a reconvolution method provided by the FluoTime interface, using the IRF obtained from excitation pulses scattered on a colloidal silica aqueous dispersion (LUDOX).

### 2.17 Transient Absorption (TA)

Femtosecond transient absorption (fsTA) spectra were measured with a pump–probe experimental setup Helios (Ultrafast Systems, LLC, USA) in combination with an ultrafast laser source consisting of Ti-Sapphire mode-locked laser Mantis that seeded a regenerative amplifier Legend Elite (Coherent, USA), yielding laser pulses with a wavelength centered at 800 nm and power of about 2 W at a 1 kHz repetition rate. Excitation pulses were derived from the optical parametric amplifier TOPAS (Light Conversion, Lithuania). The spectral time evolution was probed by white light pulses generated in a sapphire crystal. Laser pulses of both pump and probe laser beams were linearly polarized with mutual orientation of polarization at 55° (magic angle) to suppress the rotational depolarization effect on the observed time evolution of the fsTA signal. The diameter (1/*e* ^2^) of the Gaussian pump beam profile was ∼780 μm. The probe beam was elliptically shaped with axes ∼210 and ∼330 μm, respectively. The excitation was at the wavelength of 650 nm, with pump pulse energy adjusted to 400 nJ/pulse. The delay line allowed measurements of the TA time evolution for up to 6 ns.

For the measurements in time delays exceeding the nanosecond scale, the setup used for the femtosecond transient absorption described above was modified by a home-designed setup consisting of a NdYAG laser (Surelite SL I-10, Continuum, USA) as a pump source, operating at a repetition rate of 10 Hz and equipped with a frequency doubler. Probe light was generated from the electronically synchronized output of the femtosecond amplifier (800 nm, Legend Elite, Coherent, USA) operating at 20 Hz. This synchronization facilitated an effective time-resolved optical absorption measurement spanning the nanosecond to hundreds of microseconds range.^48^

Spectra were measured in air on sample solutions filled in a 2 mm optical path quartz cuvette under magnetic stirring, at concentration 0.1 mg mL^–1^. The spectral evolution was analyzed using Glotaran software providing evolution associated difference spectra with their characteristic lifetimes.

## 3 Results and Discussion

### 3.1 Synthesis and Characterization of dTPP, Polymer Precursors, and Conjugates

All dTPPs were prepared from mono carboxyl acid-substituted TPP-COOH. **D1** and **D2** were obtained by the attachment of aliphatic or aromatic oxo-alcohol to introduce the oxo group suitable for the attachment to the polymer precursors via a pH-sensitive hydrazone bond, and **D3** resulted from TT attachment to the parent photosensitizer, intended for stable amide conjugation. We confirmed the successful formation of **D1-D3** by MALDI analysis. Starting from the parent molecule TPP-COOH (molecular mass discussed in Section 2.2), the observed mass increases corresponded well with expected derivatives (calculated molecular masses: 742.9 for **D1**, 790.9 for **D2**, and 759.9 for **D3**; found molecular masses by MALDI: 742.3 for **D1**, 790.3 for **D2**, and 760.2 for **D3**).

Linear polymer precursors were synthesized by the controlled RAFT polymerization to achieve the narrow dispersity of molecular weights and ensure the molecular weight is below the limit of glomerular filtration.^49^ Both **P1** and **P2** had suitable molecular weights and dispersity for the porphyrin sensitizer delivery, and the content of hydrazide or amine groups distributed alongside the polymer chain was sufficient for the dTPP attachment (Table 1). Polymer precursors **P1** and **P2** showed hydrodynamic diameters (*D*_H_) around 10 and 7 nm, respectively, proving the formation of polymer random coil in aqueous solutions with the size enabling glomerular filtration via kidneys.

Attachment of dTPP, either via a hydrazone bond for **D1** and **D2** or via a stable amide bond, proceeded successfully for all three polymer conjugates and the amount of bound TPP-COOH was comparable, see Table 2. According to the LS detector analysis in the SEC in nonaqueous conditions, the attachment of dTPP did not lead to any significant change of the molecular weight nor the dispersity for all **C1**–**C3** conjugates (Figure 2). The conjugation of derivatives to the polymer precursors proceeded with high yields, namely, 88.6% for **C1**, 79.0% for **C2**, and 72.3% for **C3**.

**Figure 2:**
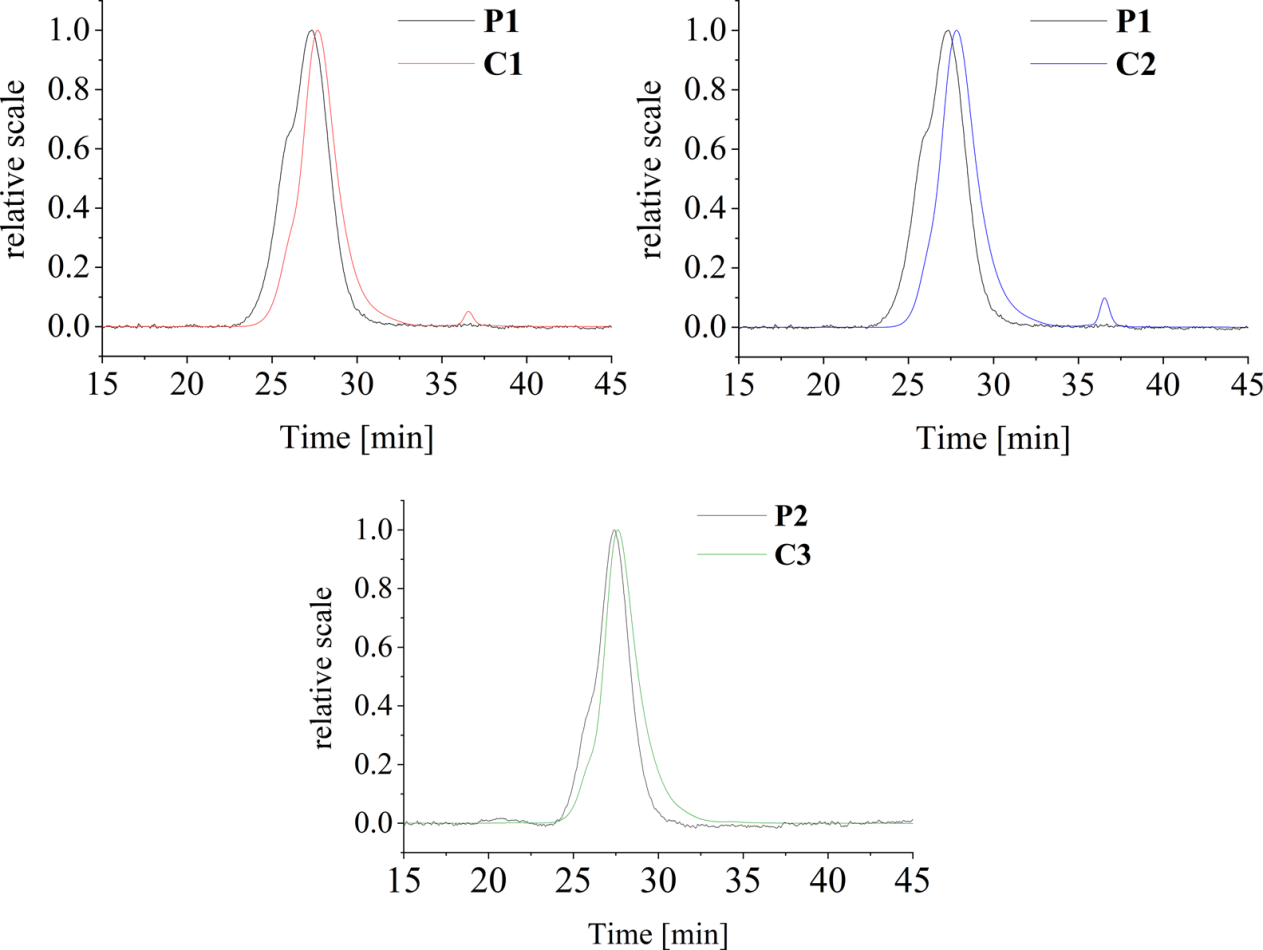
Overlapping light scattering spectra of conjugates **C1**–**C3** and their respective precursors **P1** and **P2** measured by size exclusion chromatography with DMF + LiBr (10 mM) as the mobile phase and a flow rate of 1.0 mL min^–1^.

However, the hydrodynamic diameter for all polymer conjugates increased in aqueous solutions due to the formation of supramolecular self-assembled micellar structures. The amphiphilic character after the introduction of the hydrophobic porphyrin moieties into the hydrophilic polymer structure leads to the formation of a core–shell micellar structure with an increased hydrodynamic size (Table 2). The hydrodynamic diameter of the **C1** conjugate was slightly lower, probably due to the easiest rearrangement within the micelle resulting from the more flexible aliphatic spacer.

### 3.2 Critical Micellar Concentration (CMC)

Both hydrazone conjugates **C1** and **C2** exhibited a decrease in the Soret band ratio (403 nm/423 nm band areas) upon dilution, which can be ascribed to the dissociation of stacked porphyrin units. This ratio decreased upon dilution until a critical concentration, 1.0 μg mL^–1^ for **C1** and 4.5 μg mL^–1^ for **C2**, was reached, below which it remained constant. We assume that the lower CMC for **C1** reflects an easier and more stable arrangement of **D1** in the micellar core due to the longer aliphatic spacer providing rotation and higher degree of freedom of the porphyrin units, compared to the shorter and more rigid aromatic spacer in **C2**. The conjugate **C3** showed strong dissociation even at the highest measured concentration, suggesting that the CMC is out of the chosen concentration range. This finding supports our reasoning that the shortest conjugate’s spacer in **C3** does not provide sufficient capability for easy stacking of the TPP-COOH units. The addition of 10% of human blood plasma led to even further dissociation of the **C3** conjugate, whereas it had no effects on hydrazone conjugates **C1** and **C2**. In all cases, the dissociation of the TPP-COOH units in micelles was not complete, since the Soret bands ratio did not reach zero, corresponding to 0% of stacked TPP-COOH and 100% of the monomeric porphyrin units. We speculate that upon reaching CMC, the polymer micelles dissociate into individual polymer chains, forming smaller constructs with partially stacked porphyrin moieties (Figure 3). The detailed process of CMC analysis can be found in the Supporting Information (Figure S2).

**Figure 3:**
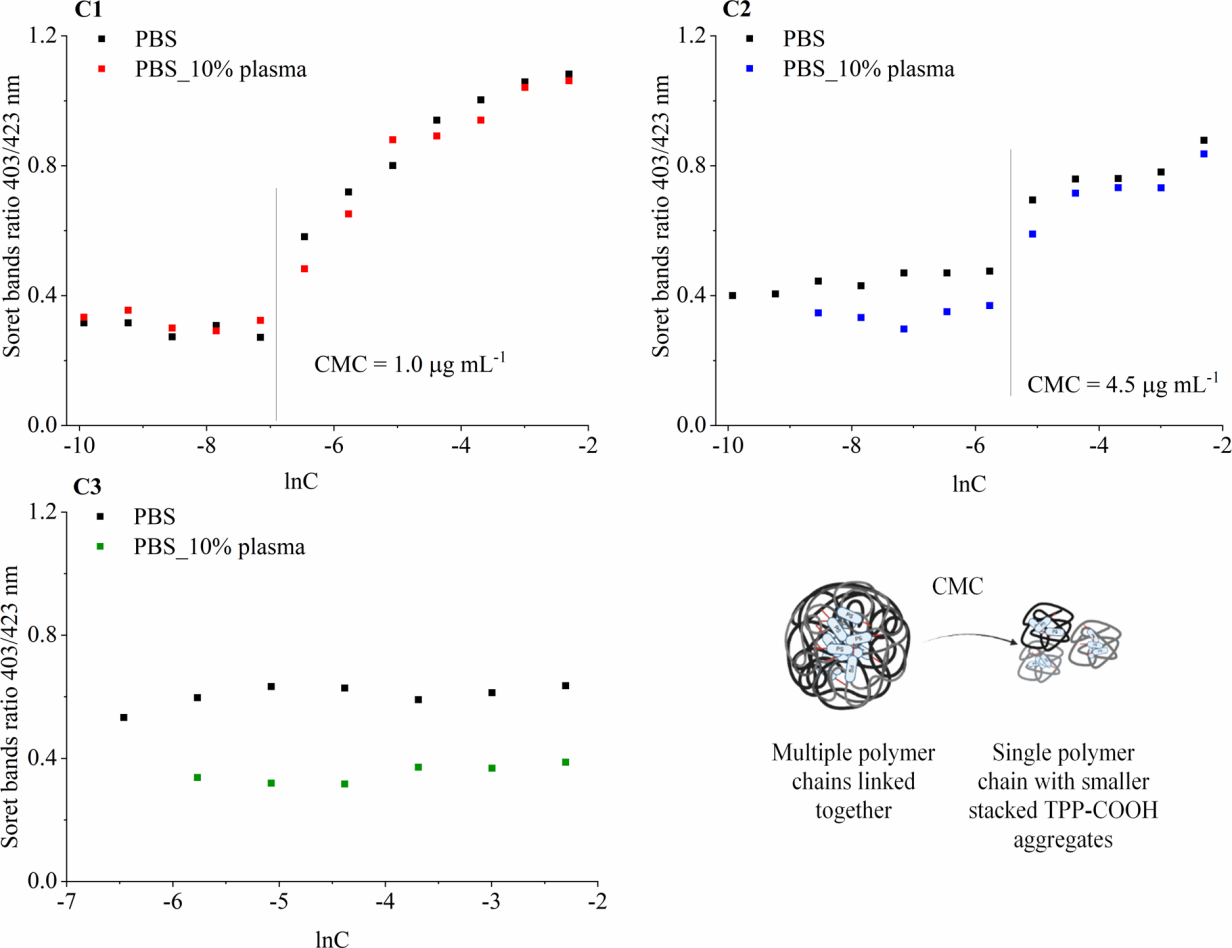
Soret band ratio of conjugates **C1**–**C3** and corresponding CMC with a graphical illustration of hypothesis. Graphical illustration was created with BioRender.com.

The interactions of polymer conjugates **C1**–**C3** and their respective precursors with blood plasma proteins were studied by using ITC. A higher polymer concentration (4.0 mg mL^–1^) than that in UV/vis experiments was used to ensure a measurable thermal response; at this level, the polymer-porphyrin conjugates are expected to be stacked. Both precursors and conjugates exhibited distinct negative enthalpy beyond the heat of dilution, characteristic of hydrophobic interactions (Figure 4 and Supporting Information Figure S3). Although the shape of the titration isotherms indicates weak binding and does not permit quantitative analysis, qualitative comparisons are possible. The **C1** conjugate, featuring a long flexible aliphatic spacer, showed the most pronounced enthalpy change. In contrast, the **C2** conjugate with a rigid aromatic spacer displayed enthalpy values similar to its precursor, while the **C3** conjugate with a very short spacer and an amide bond showed the weakest response. As all three conjugates contain nearly identical porphyrin content, the differences, which are not dramatic, likely arise from spacer rigidity affecting porphyrin accessibility for binding with proteins.

**Figure 4:**
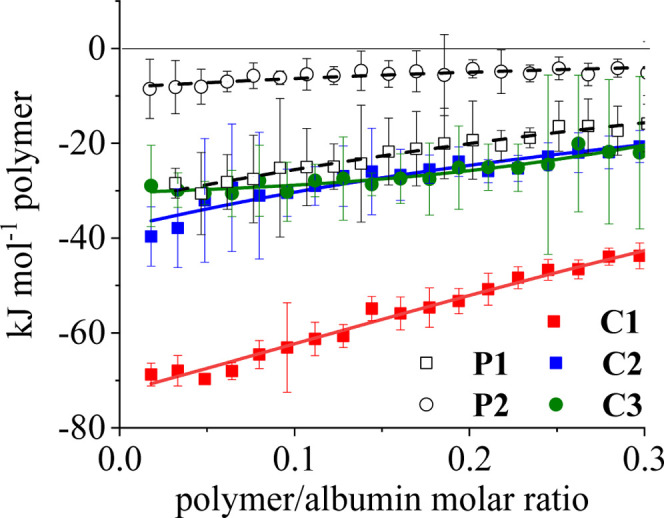
ITC measurements of conjugates **C1**–**C3** and their respective precursors **P1** and **P2**. Error bars were derived from the signal-to-noise ratio of the raw ITC data during software-based integration.

### 3.3 Release of dTPP from the Polymer Conjugates **C1** and **C2**

The release of **D1** or **D2** was evaluated in phosphate buffer pH 5.0 and pH 7.4 at 37 °C with 5% DMSO (*v*/*v*). The dTPP release was achieved by the presence of a pH-sensitive degradable spacer linking the porphyrin unit to the polymer backbone. Indeed, we observed an increased release rate at pH 5.0, mimicking the acidic lysosome environment inside the tumor cells, compared to pH 7.4, modeling the neutral blood conditions. Importantly, the structure of the used spacer significantly affected the stability of the hydrazone bond (Figure 5). In the case of aliphatic conjugate **C1**, approximately 40% of **D1** was released in pH 5.0 in 24 h, whereas only 10% of **D2** was released in the same time frame from aromatic conjugate **C2**. The similar effect of the spacer was recognized at pH 7.4. Thus, we can conclude that the aromatic spacer stabilizes the pH-sensitive hydrazone bond in its vicinity and makes the polymer conjugate significantly more stable in the biologically relevant conditions (see schematic illustration in Figure 6).

**Figure 5:**
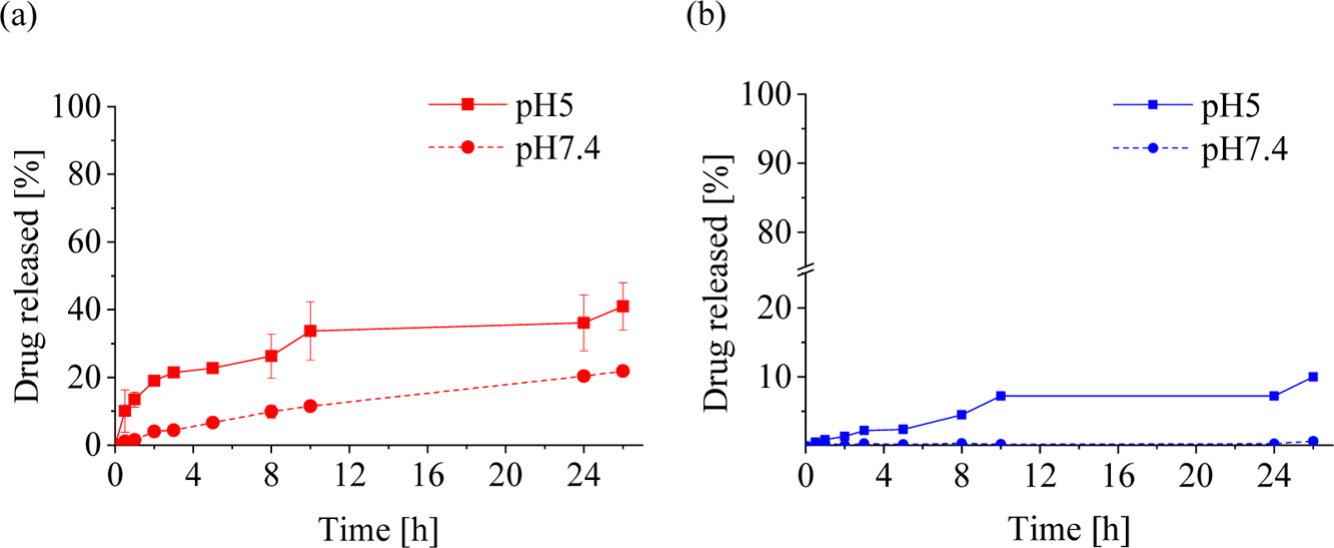
dTPP release from polymer conjugates (a) **C1** and (b) **C2** incubated in 0.1 M phosphate buffer pH 5.0 and pH 7.4 with 5% DMSO (*v*/*v*).

**Figure 6:**
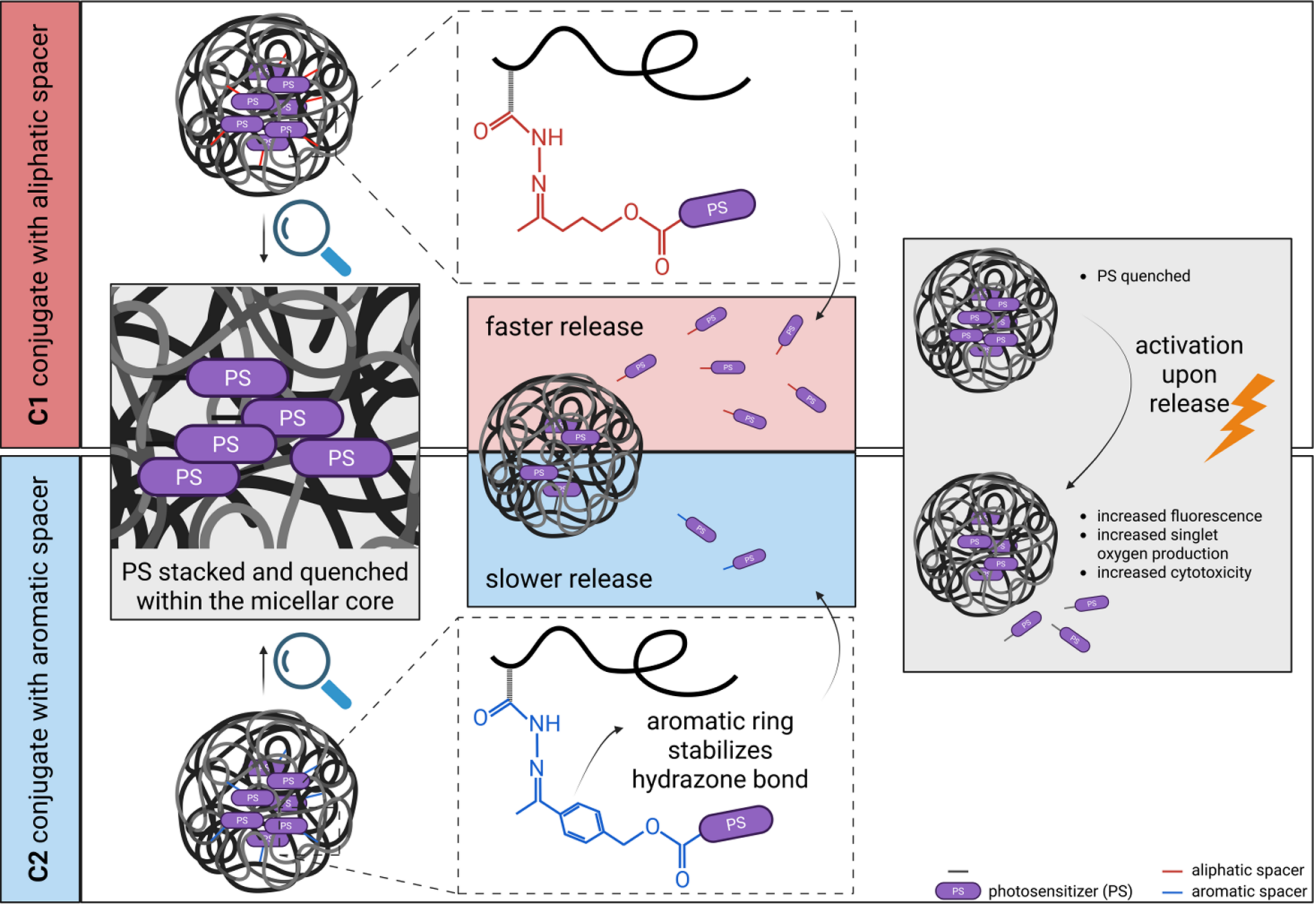
Schematic representation of spacer’s influence on release. Created with BioRender.com.

It is important to note that the PDT efficiency relies on optimal photophysical properties and the monomeric state of PS in the tumor tissues.^50^ Moreover, the extent of induced photodamage strongly depends on the PS’s localization in cells that is influenced both by the structure of PS (and resulting hydrophobicity/hydrophilicity) and the cell internalization pathway.^51^ Hydrophobic porphyrins tend to enter the cell via simple diffusion and then relocate to other membranes, resulting in high uptakes into cells in vitro, especially when maintaining relatively low concentrations.^52^ Maintaining low PS concentrations is also important in terms of aggregation.^50^ Less hydrophobic porphyrins, as well as bigger molecules and polymeric micelles, are internalized by endocytosis, typically followed by deposition in lysosomes.^50,52,53^ Binding of TPP-COOH derivatives to the polymer can therefore ensure the prolonged presence of its monomeric state, while simultaneously, introducing a pH-degradable hydrazone bond can provide slow and controlled release of the porphyrin sensitizer in its monomeric state, maintaining low porphyrin concentrations with low aggregation-induced undesired effects.

### 3.4 Micelle Formation by Fluorescence Spectroscopy

The fluorescence properties and the imaging potential of conjugates **C1**–**C3** containing 5.0 μg mL^–1^ TPP-COOH in the presence or absence of Tween-20 or SDS (Figure 7) were investigated in details. In order to minimize the inner filter effect (IFE)^54^ and to enable comparison between individual measurements, excitation wavelength was set to 514 nm and emission wavelength to 720 nm. The Lakowicz method was applied for corrections of measured fluorescence intensities in order to minimize the absorption at excitation wavelength.^55^

**Figure 7:**
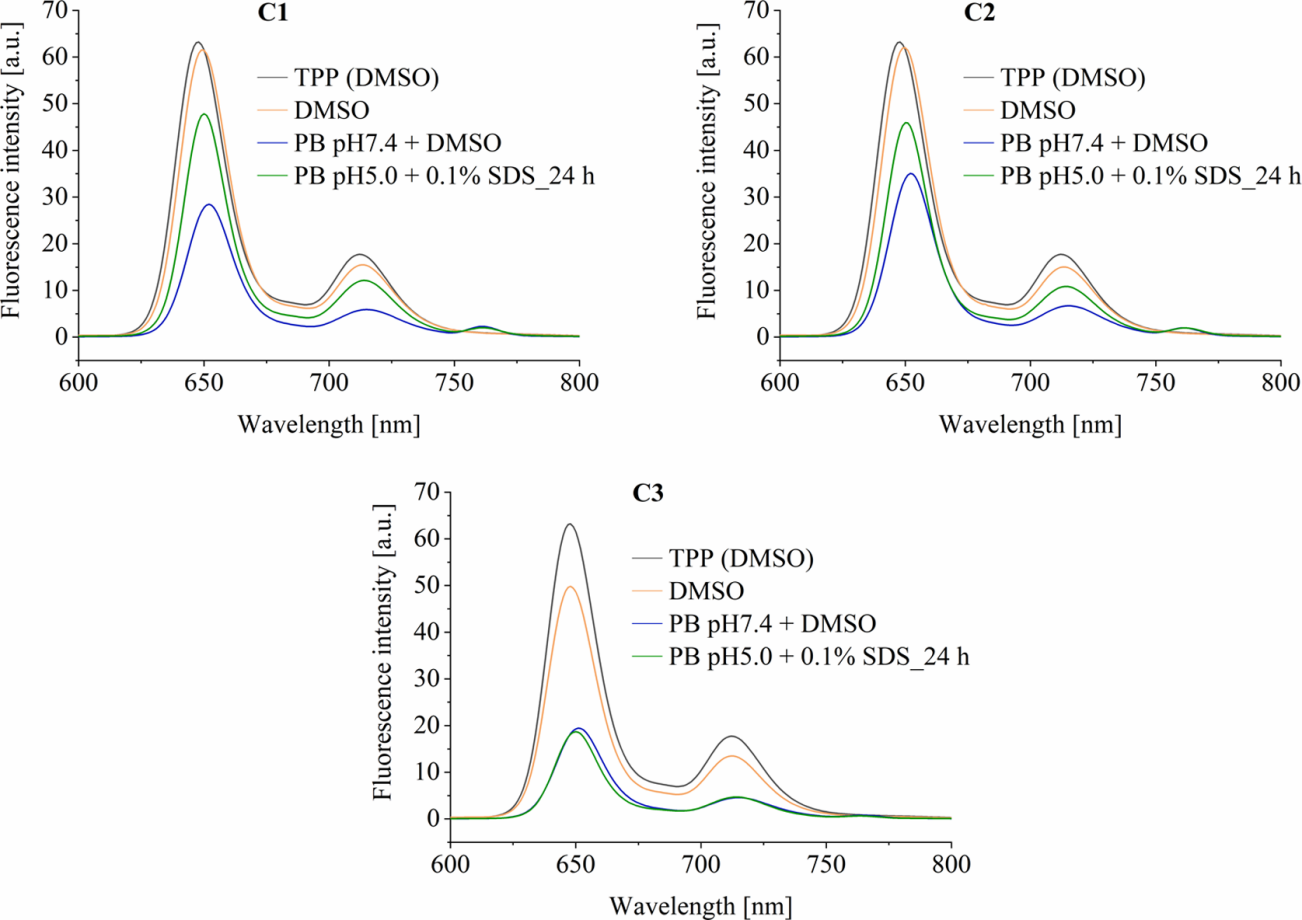
Fluorescence spectra of conjugates **C1**–**C3** in DMSO and PB pH 7.4 and after 24 h incubation in PB pH 5.0, compared to pure TPP-COOH in DMSO. All the solutions were prepared at 0.005 mg mL^–1^ TPP-COOH or equivalent and excited with λ = 514 nm. 5 % DMSO (*v*/*v*) was added for dissolution.

All conjugates in DMSO had fluorescence spectra comparable to those of pure TPP-COOH in the same solvent. It indicates that the covalent binding alongside the polymer chain keeps the respective TPP-COOH units separated with no sign of fluorescence self-quenching. The micelle formation in phosphate buffer pH 7.4/5% (*v*/*v*) DMSO led to the assembling of the porphyrin units in the hydrophobic core and partial fluorescence quenching (see Figure S4a Supporting Information). The addition of surfactants, such as SDS or Tween-20, led to the disruption of formed micelles and the increase of the fluorescence intensities to the original values comparable with the fluorescence intensities of pure TPP-COOH in DMSO (Figure S4b,c in the Supporting Information). To evaluate the influence of the porphyrin release from the conjugates on the fluorescence intensity, the conjugates were incubated at 37 °C for 24 h in phosphate buffer pH 5.0 with 5% (*v*/*v*) DMSO, with the presence of SDS for micelle disruption (Figure 7), mimicking the acidic environment of tumor tissues. Unlike for **C3**, increased fluorescence after incubation in phosphate buffer pH 5.0 compared to pH 7.4 was observed for conjugates **C1** and **C2** with a cleavable hydrazone bond. The higher intensity increase in the case of aliphatic conjugate **C1** could be ascribed to the higher porphyrin release from this conjugate. We hypothesize that such behavior would cause stimuli-based increase of the fluorescence within the tumor and as such could be used for tumor visualization as well. In this context, the presented polymer systems are particularly interesting for theranostics application. Upon conjugation of TPP-COOH to the polymer and subsequent micelle formation in aqueous solution, fluorescence is strongly quenched. As a result, these systems are expected to exhibit minimal fluorescence during systemic circulation while fluorescence is restored following TPP release and micelle disruption at the target site. We therefore propose that these systems hold potential for combining high therapeutic efficacy with fluorescence-based imaging of the tumor mass.

### 3.5 Photophysical Characterization of Polymer Conjugates **C1–C3**

The fluorescence kinetics of all three polymer conjugates **C1**–**C3** in DMSO air-saturated solutions and excited at the wavelength 590 nm corresponding to the Q_*x*_(1,0) porphyrin electronic state are shown in Figure S5 in the Supporting Information and compared to the fluorescence decay of TPP-COOH in DMSO solution as a reference. The time course of the emission from the S_1_ state recorded at 655 nm shows a single exponential decay with a lifetime of 11.1 ± 0.1 ns. There was no difference in the fluorescence kinetics observed between the samples showing that the electronic structure of the chromophore molecule is not affected by its attachment to the polymer chain. Within the experimental error, the lifetime did not change with concentration. It remained the same for all solutions with a concentration lower than 200 μg mL^–1^. On the other hand, the lifetime was sensitive to the dielectric constant of the solvent; it decreased to 9.5 ± 0.2 ns for TPP-COOH in acetone solutions, and it was slightly higher in the case when PBS was used as a solvent, reaching about 12 ns.

Transient absorption difference spectra at the ultrashort time scale (fsTA) were recorded on air-saturated DMSO solutions under the excitation at 650 nm, i.e., at the S_1_(0,0) band of the chromophore. This ensures that no excess energy is provided during the excitation, thus minimizing vibrational processes. The TA of conjugate **C1** is presented in Figure 8a together with the steady-state absorption and emission spectra. The TA spectrum at a 1 ps delay consists of a bleach (negative signal) and a positive signal of the excited state absorption (ESA) bands. The negative feature at 655 nm and the dips in the spectrum over the measured wavelength range are the result of the depletion of the ground state population upon photoexcitation. Based on the comparison with the steady-state fluorescence spectra, the small valley at 716 nm observed in the TA spectrum recorded at 1 ps delay time can be attributed to the stimulated emission.

**Figure 8:**
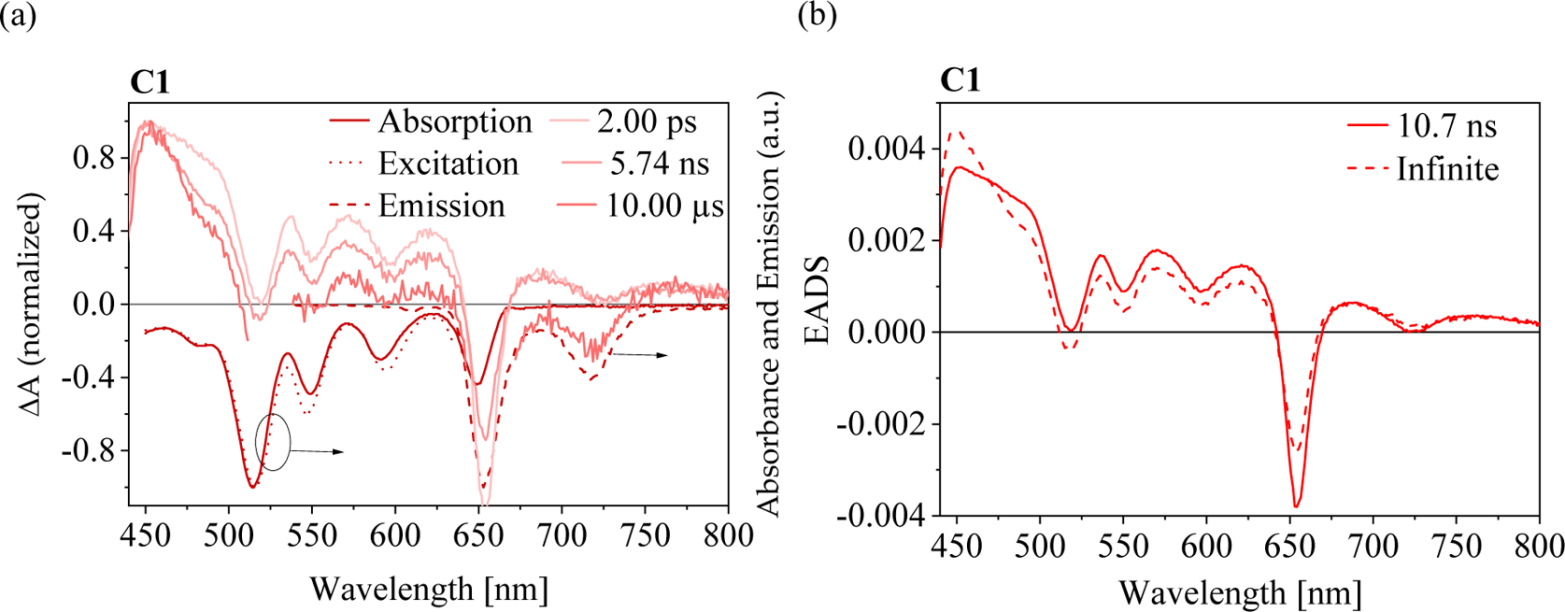
(a) TA spectra of the solutions of the conjugate **C1** in DMSO recorded at various delay times after photoexcitation (see legend), compared to the steady-state absorption and emission spectra (dot and dash lines, respectively, plotted in negative values); (b) EADS spectra obtained by the analysis with Glotaran. Excitation at 650 nm with a pump power of 400 nJ/pulse.

There were no observable differences in the TA time evolution detected between **C1**, the other conjugates, and the reference TPP-COOH in the picosecond time scale. This is best documented by their TA and evolution associated difference spectra (EADS) in the Supporting Information (Figure S6) and by the time course of the difference absorbance recorded at 570 nm (see Figure 9a). For all the compounds, the kinetics at a short time delay lack any decay features expected in the picosecond range due to interactions of chromophore TPP molecules with the solvent. The data sets can be well-fitted with two spectral components presented as EADS in Figure 8b. Due to the similarity to the fluorescence lifetime, the EADS component with the lifetime 10.6 ± 2 ns can be assigned to the ESA of the singlet S_1_ state, and the component nondecaying in the time frame of the experiment (marked as infinite) was suggested to originate from the absorption of triplets.

**Figure 9:**
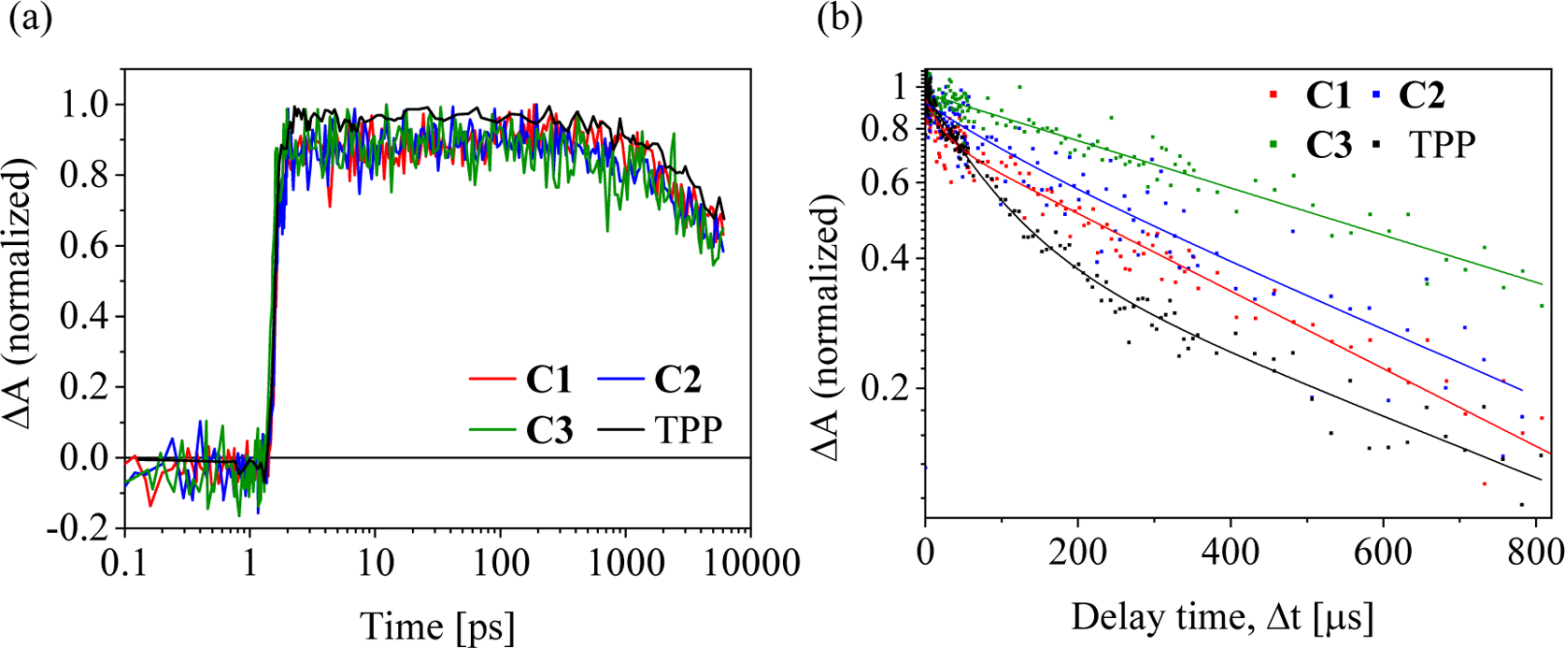
Time course of the TA of TPP-COOH and conjugates **C1**–**C3** in DMSO, concentration 0.1 mg mL^–1^ recorded on a (a) picosecond to nanosecond time scale (excitation at 650 nm, pump power 400 nJ/pulse, difference absorbance (Δ*A*) at 570 nm) and (b) longer time scale (excitation at 532 nm, pump power 1 μJ/pulse, difference absorbance at 450 nm).

To assess the spectral evolution at a longer delay time after photoexcitation, the TA experiment was performed using excitation with nanosecond pulses at 532 nm with a pump power of about 1 μJ/pulse. The TA spectra recorded at 5 μs after photoexcitation were found to be very similar to the nondecaying EADS component of TA data from the ultrafast experiment (see Figure S7 in the Supporting Information). It confirms that this EADS, with a clear maximum at 450 nm, indeed originates from the triplet absorption. The time course of the difference absorbance at 450 nm presented in Figure 9b can be well-fitted by two-exponential decays, besides sample **C3**, where the faster component is missing. The lifetimes obtained together with the amplitudes of the corresponding exponentials are summarized in Table 3. Although the data in the microsecond range are subjected to a poor signal-to-noise ratio, it seems that the increasing lifetime of triplet states when going from **C1** to **C3** corresponds to the decreasing mobility of the chromophore attached to the polymer. Surprisingly, no singlet oxygen emission was observed even when the triplet lifetimes were relatively long but this can be explained by its very weak emission and relatively low concentration of oxygen in air-saturated DMSO at room temperature, which is about 0.46 mM.^56^ For the above reasons, the measurement of singlet oxygen phosphorescence is extremely challenging and requires very sensitive detectors. Moreover, singlet oxygen can interact with triplets in TPP, repopulating them back to first excited singlets within the process called singlet oxygen-mediated mechanism^57^ and, simultaneously, relaxing singlet oxygen back to its triplet ground state.

**Table 3:** Lifetimes and Amplitudes Obtained from the Two-Exponential Fitting of the Time Course of the TA Absorption of TPP-COOH and Conjugates **C1**–**C3** Dissolved in DMSO, Concentration 0.1 mg mL^–1^, Recorded at the Microsecond Time Scale

compound	*A*_1_	τ_1_ [μs]	*A*_2_	τ_2_ [μs]
**C1**	0.2	23	0.8	480
**C2**	0.15	120	0.85	560
**C3**			1	790
TPP-COOH	0.52	83	0.47	610

### 3.6 Singlet Oxygen Production

The singlet oxygen production by the polymer conjugates was directly evaluated in vitro by measuring the generation of O_2_(^1^Δ_g_) after light irradiation. At a physiological aqueous solution of pH 7.4, no apparent O_2_(^1^Δ_g_) signal was detected; compare Figure 10 (left) and Figure S8 in the Supporting Information showing the dark O_2_(^1^Δ_g_) signal given by the impurities in the sample. However, a strong O_2_(^1^Δ_g_) signal was observed when Tween-20 was added to disrupt the micelle formations (Figure 10). These findings suggest that intact micelle formation during bloodstream circulation protects the host from PDT-induced cytotoxicity. In this state, the polymer conjugates exhibit reduced toxicity and improved safety during prolonged circulation before reaching the tumor site. Once accumulated in the tumor via the EPR effect, the tumor microenvironment may induce micellar disruption. Specifically, the acidic pH of tumor tissue and the activity of proteases such as cathepsin B and cathepsin K trigger cleavage of the chemical bonds between the PS and polymers. This process releases free PS and destabilizes the micelles, ensuring the efficient generation of singlet oxygen (O_2_(^1^Δ_g_)) from the conjugates upon light irradiation (PDT effect) at the tumor site. Furthermore, after internalization into the lysosomes of tumor cells, the lysosomal conditions may further promote micelle disruption and PS release. Notably, this effect is more pronounced in polymer conjugates containing an acid-sensitive hydrazone bond. This is exemplified by conjugate **C1**, which features a more flexible spacer between the polymer and TPP-COOH, and demonstrated a higher increase in O_2_(^1^Δ_g_) production after micelle disruption than **C2** (Figure 10). Future studies will further investigate the influence of pH on O_2_(^1^Δ_g_) generation and the PDT efficacy of different polymer conjugates.

**Figure 10:**
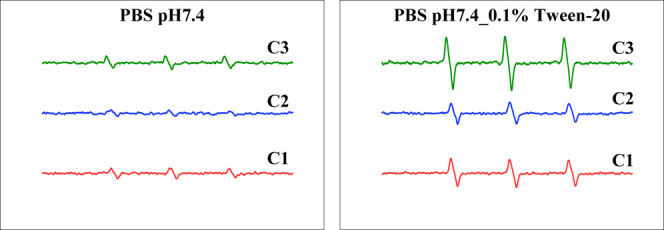
Generation of O_2_(^1^Δ_g_) from polymer conjugates **C1**–**C3** after light irradiation as detected by ESR. Polymer conjugates were dissolved in sodium phosphate buffer of pH 7.4 in the presence or absence of Tween-20, and light irradiation (90 mW cm^–2^) was carried out using a xenon light of 400–700 nm, for 300 s. O_2_(^1^Δ_g_) generated was captured by 4-oxo-TEMP, and the triplet 4-oxo-TEMPO signal due to O_2_(^1^Δ_g_) was detected by ESR spectra. See text for details.

### 3.7 In Vitro Cytotoxicity

The cytotoxicity, determined as IC_50_ values, of free TPP-COOH and conjugates **C1**–**C3** was evaluated with or without light illumination (λ = 420 nm, 5 min, 1.0 J cm^–2^). Obtained IC_50_ values are summarized in Table 4. Upon measuring dark cytotoxicity (without illumination), we found TPP-COOH is relatively toxic to the cells, with IC_50_ ∼1 μM, while all the conjugates exhibited no toxicity up to 50 μM. Thanks to enclosing cytotoxic TPP-COOH within the micellar core of conjugates **C1**–**C3**, no harm will be caused to normal tissues/cells, ensuring safety during circulation. Upon illumination, the cytotoxicity of free TPP-COOH as well as all conjugates significantly increased, while the conjugates exhibited lower cytotoxicity compared to free TPP-COOH. The pronounced cytotoxicity of free TPP-COOH, especially the dark cytotoxicity, is unlikely to be attributable to the solvent (DMSO), as its final concentration was below 1%, a level generally considered noncytotoxic. Instead, we attribute this difference to the distinct cellular uptake mechanisms between low-molecular-weight TPP-COOH and its polymer conjugates. This phenomenon is commonly observed in polymeric nanomedicines, which typically demonstrate reduced intracellular uptake and, consequently, lower cytotoxicity compared with their free drug counterparts.^39–41^ Notably, the **C1** conjugate displayed the highest cytotoxicity (lowest IC_50_), followed by **C2**, while **C3** exhibited the weakest activity. This trend correlates well with the dTPP release profiles shown in Figure 5. Collectively, these findings support the assumption that the efficient release of the free drug from the polymer carrier is critical for maximizing the PDT efficacy of the conjugates.

**Table 4:** IC_50_ Values Determined by the MTT Assay on Mouse Colorectal C26 Cells for Free TPP-COOH and Conjugates **C1**–**C3**

		TPP-COOH	**C1**	**C2**	**C3**
IC50 [μM] TPP-COOH eq	no illumination	0.86 ± 0.22	>50	>50	>50
	illumination	0.003 ± 0.0005	0.03 ± 0.002	0.15 ± 0.03	0.30 ± 0.04

We can conclude that the pH-sensitive **C1** conjugate shows promising in vitro cytotoxic efficacy with very low off-target toxicity, which was highly decreased when compared to the free TPP. Such a polymer-PS construct thus should be able to treat the tumorous cells after the EPR-based tumor accumulation and pH-sensitive release of the TPP.

In addition, when compared with other photosensitizers (PSs) previously employed for the development of polymeric PSs—such as zinc protoporphyrin IX (ZnPP),^41^ pyropheophorbide-a (PyF-a),^39^ and the clinically used PDT agent temoporfin (mTHPC)^40^—TPP-COOH demonstrated much higher dark cytotoxicity and photocytotoxicity. Specifically, TPP-COOH showed stronger activity than ZnPP (dark cytotoxicity ∼32 μM; photocytotoxicity ∼0.014 μM) and PyF-a (dark cytotoxicity >10 μM; photocytotoxicity ∼0.21 μM), while its activity was comparable to that of the clinically approved mTHPC (dark cytotoxicity ∼1 μM; photocytotoxicity ∼0.0005 μM). These findings highlight the high therapeutic potential of TPP-COOH as a PDT agent and support the clinical applicability of its polymer conjugates.

## 4 Conclusions

In this work, we successfully designed, developed, and thoroughly characterized the physicochemical properties of polymer-based nanomedicines functioning as stimuli-responsive theranostics for the treatment and imaging of solid tumors. These nanomedicines were constructed by using a micelle-forming polymer-TPP conjugate, enabling pH-sensitive activation of both singlet oxygen generation and fluorescence. Safety in systemic circulation should be ensured by the self-assembly of these conjugates into stable micellar structures in aqueous environments, thereby minimizing potential side effects commonly associated with nanomedicine-based therapies. Notably, introducing either aliphatic or aromatic spacers between the TPP-COOH and the polymer backbone enhanced the flexibility of the polymer systems and promoted the stability of the micellar assemblies, an essential feature for effective drug delivery. Furthermore, the hydrazone bond enabled tumor-specific degradation of the conjugates, offering a highly efficient mechanism for the targeted release of photosensitizers under the acidic conditions typical of the tumor microenvironment. This tumor-responsive breakdown significantly enhances the therapeutic potential of the system. Overall, the hydrazone-linked polymer-TPP conjugates exhibited excellent stability, responsiveness, and functional activation, positioning them as promising candidates for next-generation anticancer theranostic applications.

## Supplementary Material



## Notes

Figure S1: MALDI-TOF mass spectra of free TPP-COOH and its respective derivatives **D1**–**D3**; Figure S2: Process of CMC evaluation based on measured UV/vis spectra of conjugates **C1** in PBS. Other conjugates, **C2**, and **C3** were subjected to the same procedure; Figure S3: ITC dilution experiment of conjugates **C1**–**C3** and their respective polymer precursors **P1** and **P2** to a pure buffer; Figure S4: Fluorescence spectra of conjugates **C1**–**C3** (a) compared with pure TPP-COOH in DMSO and 0.1 M phosphate buffer pH 7.4, (b) with or without the presence of Tween-20, or (c) sodium dodecyl sulfate (SDS). All the solutions were prepared at 0.005 mg mL^–1^ TPP-COOH or equivalent and excited with λ = 514 nm. 5 % DMSO (*v/v*) was added for dissolution; Figure S5: Fluorescence kinetics of **C1** (red), **C2** (blue), **C3** (green), and TPP-COOH (black) dissolved in DMSO, concentration 100 μg mL^–1^. Grey line: instrument response function (IRF), light blue dashed line – single exponential fit with lifetime 11.1 ns. Excitation wavelength 590 nm, emission wavelength 655 nm in all cases; Figure S6: (a) Normalized TA spectra of the solutions of conjugates **C2**, **C3**, and TPP-COOH recorded at various delay times after photoexcitation (see legend), compared to the steady-state absorption, excitation (emission at 654 nm), and emission (excitation at 419 nm) spectra, shown in negative values, and (b) EADS spectra obtained by the analysis with Glotaran; Figure S7: Comparison of the “infinite” EADS component obtained from the fsTA spectroscopy acquired in an ultrashort time scale with the TA spectra recorded at 5 μs after photoexcitation; and Figure S8: generation of O_2_(^1^Δ_g_) from polymer conjugates **C1**–**C3** without light irradiation as detected by ESR. Polymer conjugates were dissolved in PBS pH 7.4 in the presence of 0.1 % Tween-20. O_2_(^1^Δ_g_) generated was captured by 4-oxo-TEMP, and triplet 4-oxo-TEMPO signal due to O_2_(^1^Δ_g_) was detected by ESR spectra. See text for details. (PDF)

## Abbreviations

Δ*A*: difference absorbance
ACVA: 4,4′-azobis­(4-cyanovaleric acid)
AIBN: 2,2́-azobis­(isobutyronitrile)
AIBN-TTc: *S*-2-cyano-2-propyl-*S*′-ethyl trithiocarbonate
carboxyethyl-TTc-ACVA: 4-(2-carboxyethylsulfanylcarbothioylsulfanyl)-4-cyanopentanoic acid
CMC: critical micellar concentration
CTA: chain transfer agent
*Đ*: dispersity
DCM: dichloromethane
*D*_H_: hydrodynamic diameter
DIPEA: *N*,*N*-diisopropylethylamine
DLS: dynamic light scattering
DMA: *N*,*N*-dimethylacetamide
DMAP: 4-(dimethylamino)­pyridine
DMF: *N*,*N*-dimethylformamide
DMSO: dimethyl sulfoxide
dTPP: derivative of TPP-COOH
EADS: evolution associated difference spectra
EDC: *N*-(3-(dimethylamino)­propyl)-*N*′-ethylcarbodiimide hydrochloride
EPR: enhanced permeability and retention
ESA: excited state absorption
ESR: electron spin resonance
EtOH: ethanol
FG: functional group
fsTA: femtosecond transient absorption
HPLC: high-performance liquid chromatography
HPMA: *N*-(2-hydroxypropyl)­methacrylamide
HRMS: high-resolution mass spectrometry
IRF: instrument response function
Ma-Ahx-NHNH-Boc): *N*-(*tert*-butoxycarbonyl)-*N*′-(6-methacrylamidohexanoyl)­hydrazine
Ma-AP-NH-Boc: *N*-(3-*tert*-butoxycarbonyl-aminopropyl)­methacrylamide
MALDI: matrix-assisted light desorption/ionization
MeOH: methanol
*M*_n_: number-average molecular weight
*M*_w_: weight-average molecular weight
NMR: nuclear magnetic resonance
PB: phosphate buffer
PBS: phosphate buffer saline
PDT: photodynamic therapy
PS: photosensitizer
PyF: pyropheophorbide-a
RAFT: radical reversible addition–fragmentation chain transfer
ROS: reactive oxygen species
SD: standard deviation
SDS: sodium dodecyl sulfate
SEC: size exclusion chromatography
TA: transient absorption
*t*-BuOH: *tert*-butanol
TCSPC: time-correlated single photon counting
TFA: trifluoroacetic acid
TNBSA: 4,6-trinitrobenzene-1-sulfonic acid
TPP: tetraphenylporphyrin
TT: 2-thiazoline-2-thiol
V-70: 2,2′-azobis­(4-methoxy-2,4-dimethylvaleronitrile)
