# Supplementary material for: Polymeric Nanocarriers of a Monosubstituted Tetraphenylporphyrin Sensitizer Intended for Photodynamic Therapy and Tumor Imaging
Source: ACS Omega. 2025 Nov 10;10(46):56201–16. doi: 10.1021/acsomega.5c07910 (PMC12658679; doi:10.1021/acsomega.5c07910)
Supplement: Supplementary file 1 [file ao5c07910_si_001.pdf]

Supporting Information

Polymeric nanocarriers of monosubstituted tetraphenylporphyrin sensitizer intended for photodynamic therapy and tumor imaging

Alžběta Turnovská[1], Jan Hynek[2], Marina Rodrigues Tavares[1], Muhammed Arshad Thottappali[1], Shanghui Gao[3], Volodymyr Lobaz[1], Jiří Pfleger[1], Jun Fang[3], Kamil Lang[2] and Tomáš Etrych[1]*

[1] Institute of Macromolecular Chemistry, Czech Academy of Sciences, Prague, Czech Republic

E-mail: turnovska@imc.cas.cz, etrych@imc.cas.cz

[2] Institute of Inorganic Chemistry, Czech Academy of Sciences, Prague, Czech Republic

E-mail: lang@iic.cas.cz

[3] Laboratory of Microbiology and Oncology, Faculty of Pharmaceutical Sciences, Sojo University, Kumamoto, Japan

E-mail: fangjun@ph.sojo-u.ac.jp

2. Materials and Methods

2.8. Size exclusion chromatography (SEC)

A high-performance liquid chromatography (HPLC) Shimadzu system equipped with a photodiode array UV-VIS detector (SPD-M40, Schimadzu, Japan), a refractometric detector (Optilab, Wyatt, Germany) and an 8-angle light scattering detector (DAWN, Wyatt, Germany) was used. The analysis was performed with DMF + LiBr (10 mM) as a mobile phase on series of PSS Gram columns (Germany) with a flow rate of 1.0 mL min^{-1}. Measured data were analyzed using the ASTRA 8.1 (Wyatt) software and LabSolutions 5.106 (Schimadzu). The refractive index increment *dn*/*dc* was set at approximately 0.09 mL g^{-1}.

2.13. Release of dTPP from the polymer conjugates C1 and C2

A HPLC LC10 system, equipped with a detector SPD-M20A (Schimadzu, Japan) and COSMOSIL 5C4-AR-300 column (Nacalai Tesque, INC. Kyoto Japan) was used. Water/acetonitrile 95/5 with 0.1 % TFA and DMF/acetonitrile 50/50 with 0.1 % TFA was used as an eluent with a gradient 50-100 % at a flow rate of 2.5 mL min^{-1}.

2.18. Matrix assisted light desorption/ionization (MALDI)

The purity of TPP-COOH and derivatives **D1-D3** was evaluated using matrix assisted light desorption/ionization (MALDI). The dried droplet method was used to prepare samples for MALDI–TOF mass spectrometry measurements. Solutions of the sample (10.0 mg mL^{-1}) and matrix DCTB (trans-2-[3-(4-tert-Butylphenyl)-2-methyl-2-propenylidene]malononitrile; 10.0 mg mL^{-1}) in DCM were mixed in a volume ratio 4:20. 1.0 µL of the mixture was deposited on the ground-steel target. Then, MALDI-TOF mass spectra were acquired with an UltrafleXtreme TOF mass spectrometer (Bruker Daltonics, Bremen, Germany) equipped with a 2000 Hz smartbeam-II laser (355 nm) in the positive ion reflectron mode. Panoramic pulsed ion extraction and external calibration were used for molecular weight assignment.

3. Results and discussion

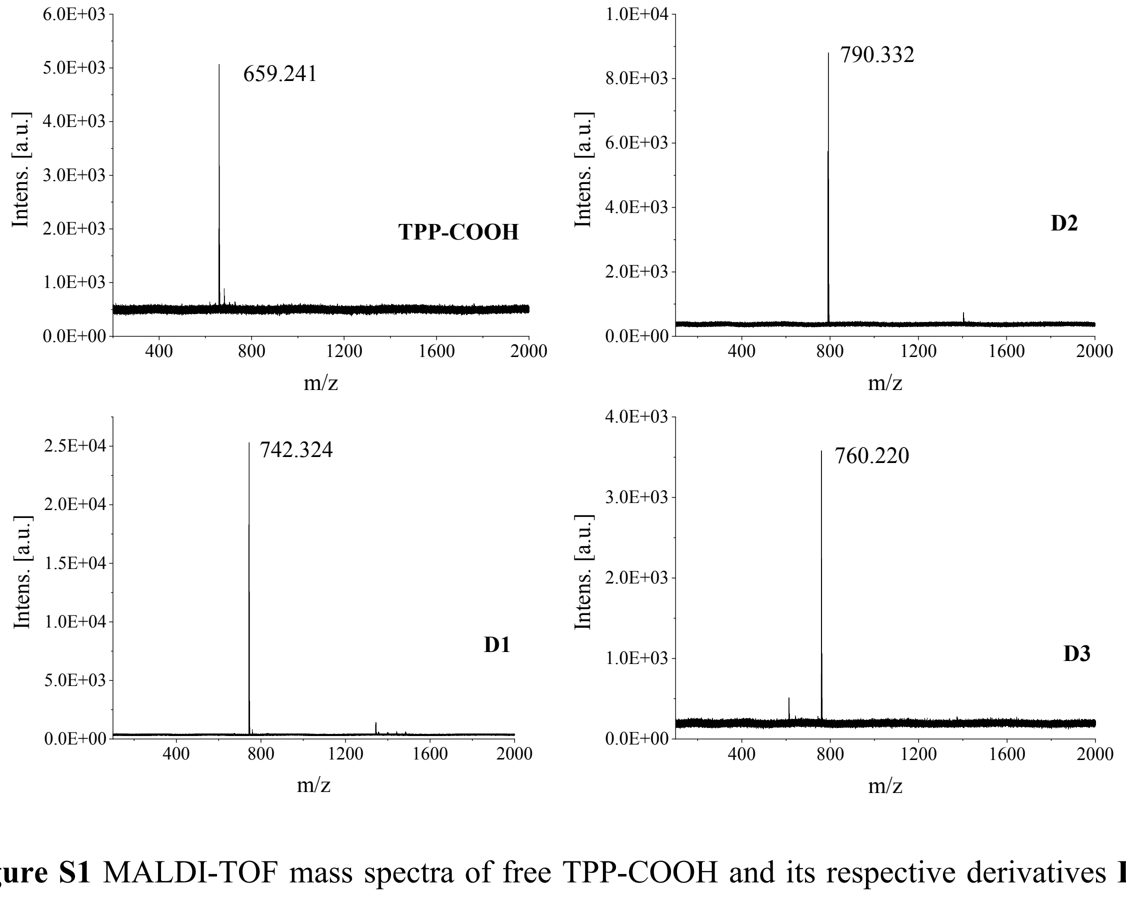

Figure S1 MALDI-TOF mass spectra of free TPP-COOH and its respective derivatives **D1-D3**.

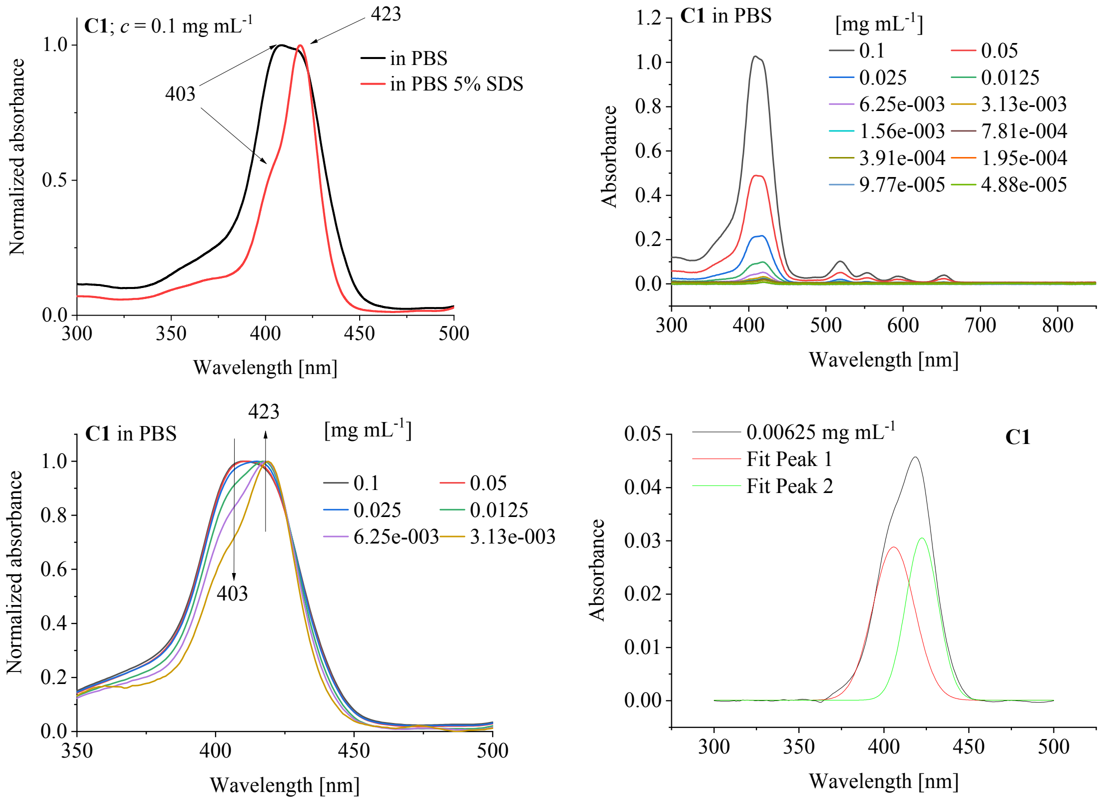

Upon addition of SDS (5 wt% in PBS), the intensity of signal at 423 nm, originating from dissociation of porphyrin aggregates after micellar disruption, increased. → We measured spectra of **C1** with different concentrations → Observed change in Soret band peak ratio upon concentration decrease

Figure S2 Process of CMC evaluation based on measured UV/VIS spectra of conjugate **C1** in PBS. Other conjugates, **C2** and **C3**, were subjected to the same procedure.

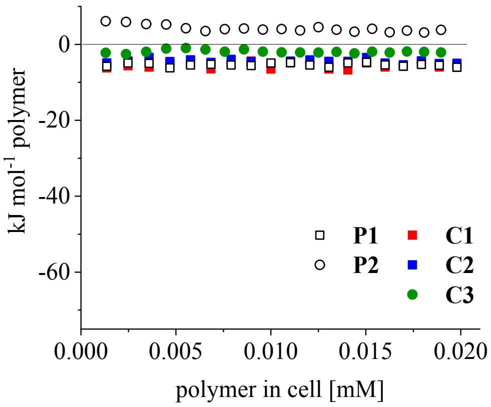

Figure S3 ITC dilution experiment of conjugates **C1-C3** and their respective polymer precursors **P1** and **P2** to a pure buffer.

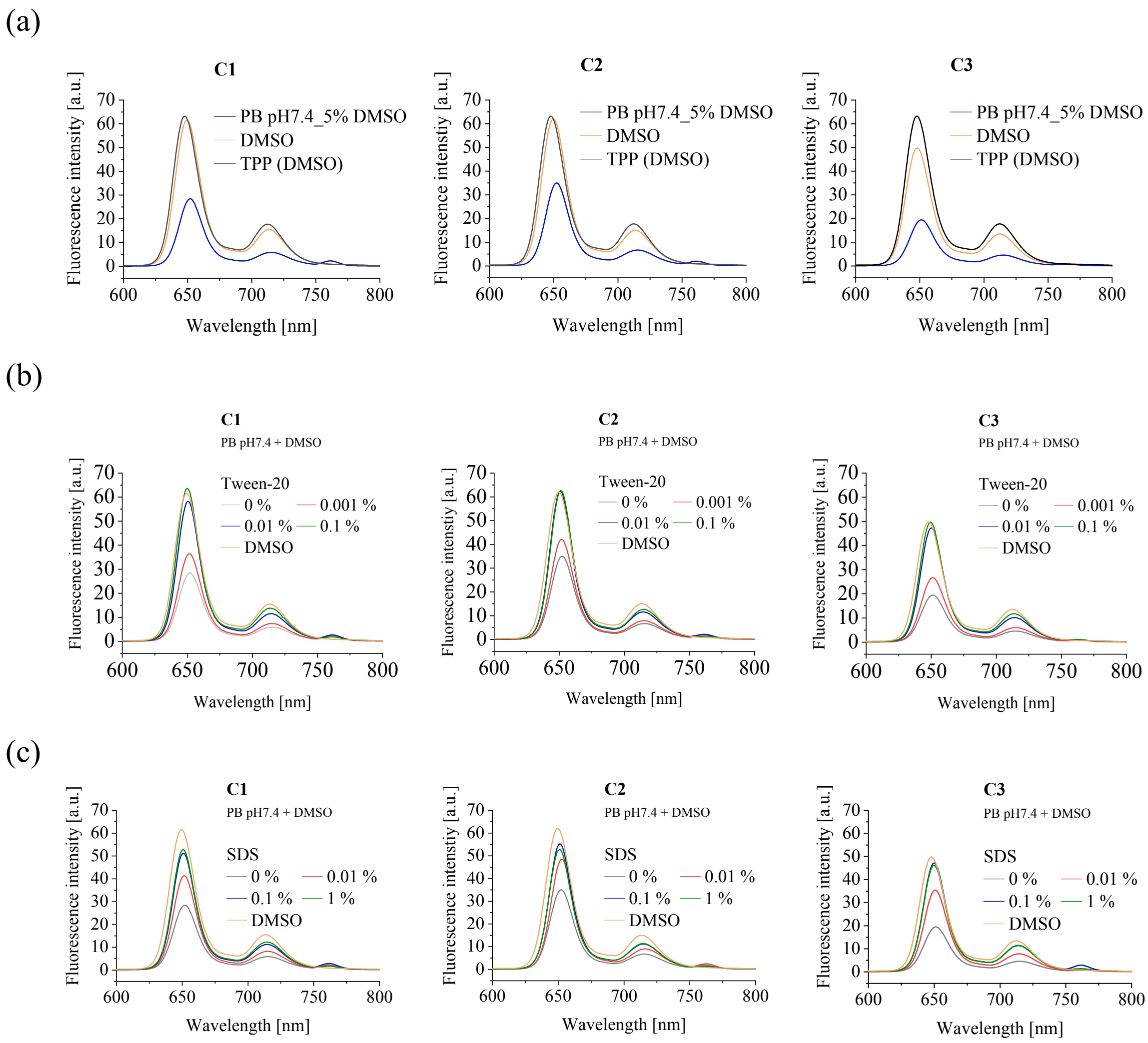

Figure S4 Fluorescence spectra of conjugates **C1** - **C3** (a) compared with pure TPP-COOH in DMSO and 0.1 M phosphate buffer pH 7.4, (b) with or without the presence of Tween-20, or (c) sodium dodecyl sulphate (SDS). All the solutions were prepared at 0.005 mg mL^{-1} TPP-COOH or equivalent and excited with λ = 514 nm. 5 % DMSO (*v/v*) was added for dissolution.

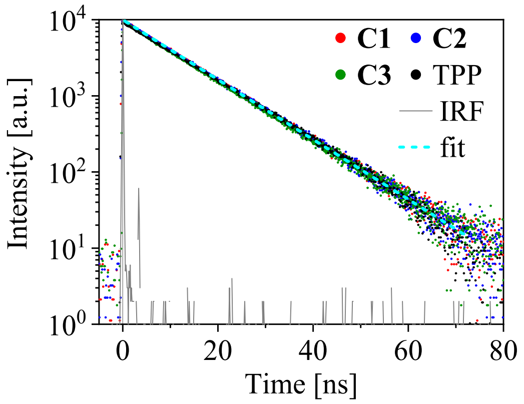

Figure S5 Fluorescence kinetics of **C1** (red), **C2** (blue), **C3** (green) and TPP-COOH (black) dissolved in DMSO, concentration 100 µg mL^{-1}. Grey line: instrument response function (IRF), light blue dashed line – single exponential fit with lifetime 11.1 ns. Excitation wavelength 590 nm, emission wavelength 655 nm in all cases.

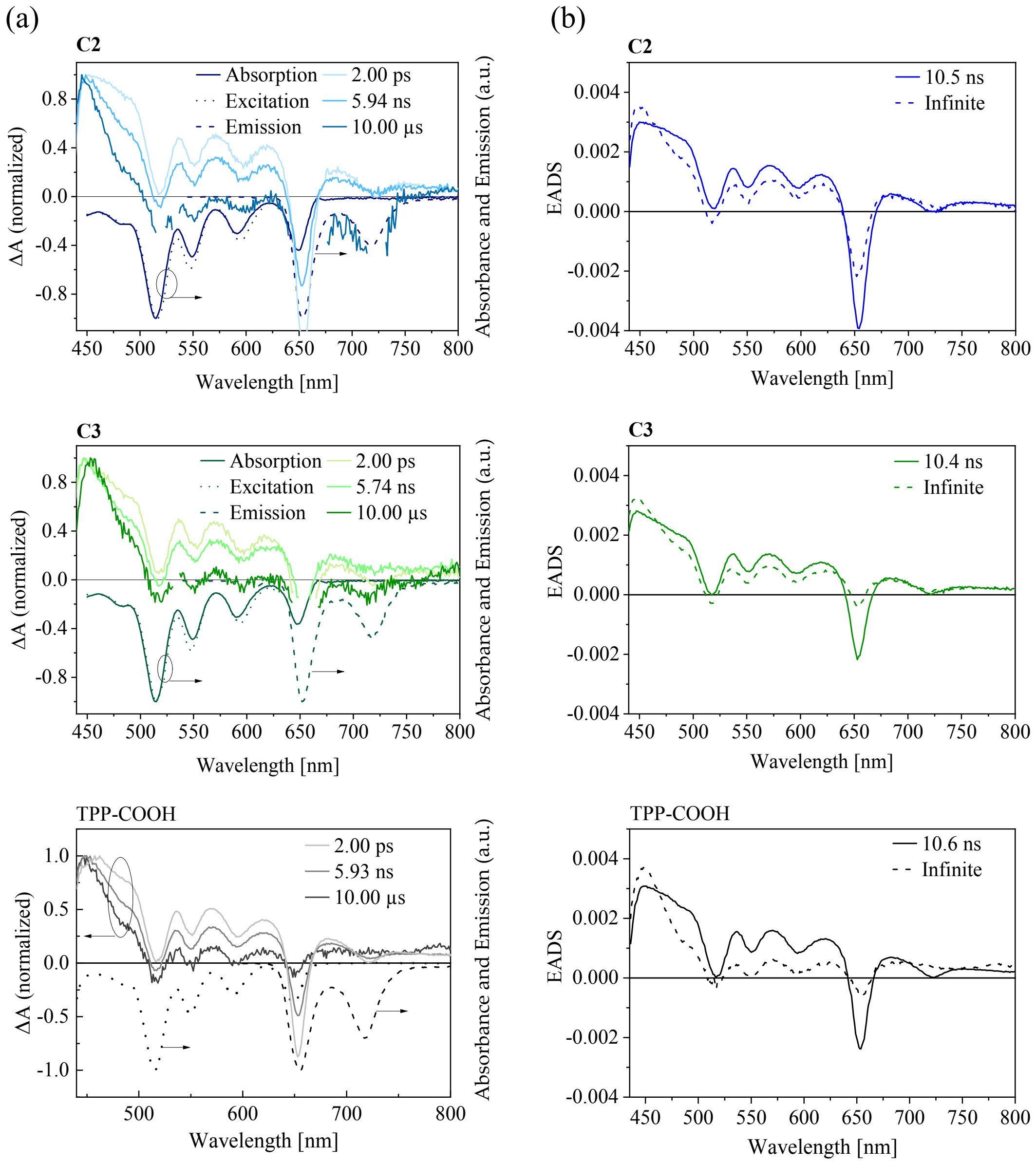

Figure S6 (a) Normalized TA spectra of the solutions of conjugates **C2**, **C3** and TPP-COOH recorded at various delay time after photoexcitation (see legend), compared to the steady-state absorption, excitation (emission at 654 nm) and emission (excitation at 419 nm) spectra, shown in negative values. (b) EADS spectra obtained by the analysis with Glotaran.

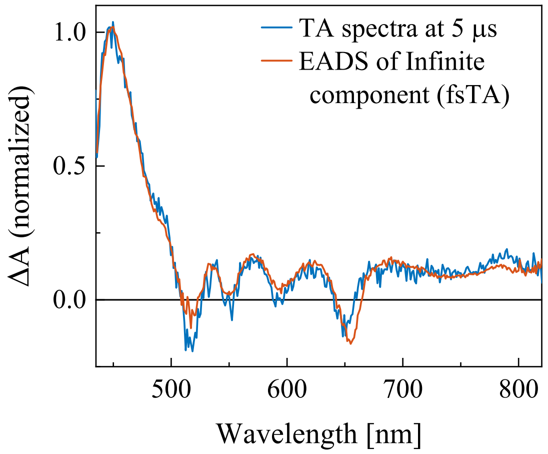

Figure S7 Comparison of the "infinite" EADS component obtained from the fsTA spectroscopy acquired in ultrashort time scale with the TA spectra recorded at 5 μs after photoexcitation.

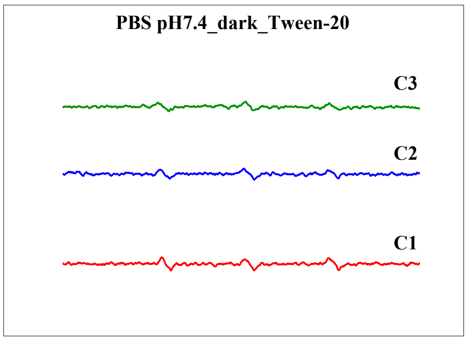

Figure S8 Generation of $O_2(^1\Delta_g)$ from polymer conjugates **C1-C3** without light irradiation as detected by ESR. Polymer conjugates were dissolved in PBS pH 7.4 in the presence of 0.1 % Tween-20. $O_2(^1\Delta_g)$ generated was captured by 4-oxo-TEMP, and triplet 4-oxo-TEMPO signal due to $O_2(^1\Delta_g)$ was detected by ESR spectra. See text for details.
